# Glucose levels are associated with mood, but the association is mediated by ratings of metabolic state

**DOI:** 10.1016/j.ebiom.2025.106035

**Published:** 2025-12-08

**Authors:** Kristin Kaduk, Marie Kaeber, Anne Kühnel, María Berjano Torrado, Melina Grahlow, Birgit Derntl, Nils B. Kroemer

**Affiliations:** a Department of Psychiatry and Psychotherapy, Tübingen Center for Mental Health, University of Tübingen, Tübingen, Germany; b Section of Medical Psychology, Department of Psychiatry and Psychotherapy, Faculty of Medicine, University of Bonn, Bonn, Germany; c German Center for Mental Health (DZPG), Partner Site Tübingen, Germany; d German Center for Diabetes Research (DZD), Neuherberg, Germany

**Keywords:** Continuous glucose monitoring, Interoception, Ecological momentary assessment, Longitudinal

## Abstract

**Background:**

Hunger is commonly linked to negative mood, and mood shifts are believed to arise from sensing the body's internal state. However, it remains unclear whether this link is driven by subconscious effects of circulating glucose levels or by consciously sensed metabolic states. Here, we test whether glucose levels directly influence mood or indirectly via subjective ratings of metabolic state.

**Methods:**

In this observational cohort study, 90 healthy adults (female = 46; male = 44) were continuously monitored throughout the day using interstitial glucose sensors for four weeks while completing ecological momentary assessments up to twice per day (EMA; *M* = 48 assessments per participant) to rate mood and perceived metabolic states.

**Findings:**

As expected, hungry participants reported lower mood, and metabolic state ratings were associated with glucose levels. Although glucose levels were associated with mood, the metabolic state ratings mediated this association. Individual differences reflecting metabolic health (i.e., BMI and insulin resistance) did not affect the interaction between glucose and metabolic state ratings on mood. Notably, individuals with higher interoceptive accuracy had fewer fluctuations in mood ratings.

**Interpretation:**

We conclude that hunger-related mood shifts depend on conscious sensing of the body's internal state instead of acting subconsciously. Our study highlights the relevance of considering the self-report of bodily signals in understanding mood shifts, offering new fundamental insights into mood regulation mechanisms.

**Funding:**

The study was supported by the 10.13039/501100001659German Research Foundation (DFG) grants KR 4555/7-1, KR 4555/9-1, KR 4555/10-1, and DE 2319/22-1.

**Research in context**

**Evidence before this study**

Hunger is known to affect mood, and it is assumed that this is driven by a homeostatic process guiding food intake in response to changes in glucose levels. Manipulating glucose has been linked to changes in mood in laboratory experiments, although the results have been inconsistent, particularly in individuals with metabolic disorders. Whether glucose levels influence mood directly or indirectly through conscious awareness of metabolic states in everyday life remains unresolved to date.

**Added value of this study**

By combining four weeks of continuous glucose monitoring with ecological momentary assessment in 90 healthy adults, our results show that the link between glucose levels and mood is mediated by metabolic state ratings (hunger minus satiety). In other words, lower glucose affected mood only if participants also reported being hungry. Moreover, individuals with higher interoceptive accuracy (i.e., better correspondence between glucose levels and hunger ratings) experienced fewer mood fluctuations.

**Implications of all the available evidence**

Our findings demonstrate that hunger-related mood changes depend on consciously sensed metabolic states rather than subconscious signaling of glucose levels. This supports psychological theories of emotion, emphasising the integration of bodily signals into conscious experience. Notably, considering such interoceptive processes may improve our understanding of mood regulation and explain why metabolic dysfunctions, such as obesity and insulin resistance, are often comorbid with mood disorders. Future research should extend this approach to clinical populations and examine additional physiological mediators (i.e., hormone levels and stress).

## Introduction

Hunger often precipitates mood changes, likely serving a vital function^1^^,^^2^ by signalling the body's energy demands and guiding food intake.^3^, ^4^, ^5^ Energy metabolism is crucial for mental health, with metabolic disorders linked to psychological sequelae.^6^^,^^7^ Central to this interaction between energy metabolism and mood is glucose, the body's primary energy source,^8^^,^^9^ influencing mood,^10^, ^11^, ^12^ aggression,^13^^,^^14^ and tension.^10^^,^^15^ Despite the importance of body–brain interactions for mental health and well-being, it remains unclear whether circulating glucose affects mood beyond self-reported metabolic states (hunger, satiety).

The homeostatic view states that physiological signals, such as glucose, shape hunger and mood to counteract a negative energy balance. Low glucose levels trigger hormone release (e.g., glucagon) to mobilise glucose from liver stores, inhibit insulin secretion, stimulate appetite, and facilitate eating in rats,^16^^,^^17^ and humans.^18^^,^^19^ Elevated glucose levels stimulate insulin secretion, inducing satiety, decreasing tension,^15^ and appetite.^20^ However, laboratory studies on mood-related effects of glucose,^21^ often using high-calorie drinks or foods, have yielded inconclusive results.^11^^,^^12^^,^^15^^,^^22^, ^23^, ^24^, ^25^, ^26^ Continuous glucose monitoring (CGM) enables research beyond controlled, but artificial settings.^27^^,^^28^ Combining CGM with ecological momentary assessment (EMA, real-time sampling of subjective ratings, e.g., mood, in daily life via smartphones) allows naturalistic studies of daily mood fluctuations while reducing recall biases. For example, in patients with type 2 diabetes, higher glucose levels (>180 mg/dL, reflecting hyperglycemia in a state of insulin resistance) were associated with negative mood.^29^ In contrast, in healthy adolescents, a modest elevation in glucose within the physiological postprandial range (≈90–130 mg/dL) was related to elevated mood and reduced fatigue.^30^ These diverging findings likely reflect distinct physiological mechanisms related to energy homeostasis; however, both illustrate that fluctuations in glucose levels are associated with mood. Notably, prior work has not tested whether these effects operate directly through glucose or are mediated by the subjective experience of hunger.

Hunger motivates energy intake in response to ingestion-related internal cues,^31^^,^^32^ acting as both a homeostatic signal and an allostatic signal to anticipate negative excursions in energy balance (e.g., low energy supply). In animals, metabolic states influence behaviour and brain responses. Agouti-related peptide (AgRP) neurons in the hypothalamus signal hunger's negative valence.^33^ Energy intake restores glucose levels, modulating the activity of metabolic-sensing AgRP neurons and activating the dopaminergic mesolimbic system,^34^ shifting hunger's negative valence state into positive reinforcement. In humans, hunger and satiety are intricately linked to mood.^35^^,^^36^ Hunger is related to restlessness, excitability,^35^ sadness,^37^ increased irritability, aggression, and anger,^36^^,^^38^ whereas satiety promotes satisfaction, relaxation, and calmness.^35^ Accordingly, hungry women with a healthy weight reported higher tension, anger, fatigue, and confusion, and lower vigor and esteem.^39^

Findings suggesting that hunger contributes to negative mood are consistent with the psychological construction of emotion theory.^40^^,^^41^ It posits that homeostatic processes (e.g., hunger as a signal of energy imbalance) generate core affect, shaping mood through bodily sensations and external stimuli.^1^^,^^42^^,^^43^ For example, MacCormack & Lindquist (2019)^1^ found that hunger biases perception and judgment, heightening negativity, particularly when individuals were unaware of their emotions, showing that sensing and interpretation of internal bodily signals, called interoception, is crucial.^44^, ^45^, ^46^ Instead, the psychological construction of emotion theory posits that interoception transforms physiological states (e.g., low blood sugar) into the cognitive experience of hunger by integrating bodily sensations with contextual information.^42^^,^^47^^,^^48^ Interoceptive accuracy in detecting metabolic changes, therefore, likely influences mood regulation as it arises from sensing bodily signals.

To summarise, glucose surges are associated with satiety, and hunger is linked to negative mood states. However, it remains unclear whether conscious processes reflected in subjective ratings of metabolic state mediate the association between glucose and mood. To address this gap, we conducted a four-week study combining CGM and EMA in naturalistic settings to examine the association of glucose levels and metabolic state ratings on mood (happiness and sadness). Consistent with previous findings, we expected lower glucose levels to be associated with decreased metabolic state ratings and mood. Our study shows that the association of glucose with mood was mediated by metabolic state ratings. Moreover, higher interoceptive accuracy was associated with fewer mood fluctuations, highlighting the role of interoception in mood regulation in accordance with the psychological construction of emotion theory.

## Methods

**Fig. 1:**
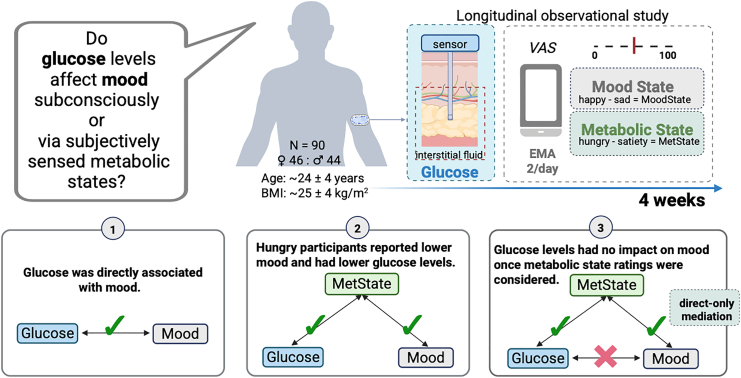
**Study and results summary.** In a 4-week observational cohort study, 90 healthy adults were continuously monitored with interstitial glucose sensors while completing ecological momentary assessments of happy, sad, hunger, and satiety. The three panels summarize the main results that the link between mood and metabolic state is mediated by subjective ratings of metabolic state, indicating indirect-only mediation. MetState, metabolic state; Glucose, glucose levels; Green arrows, significant path; red cross, non-significant path; VAS, visual analogue scale; EMA, Ecological momentary assessment; CGM, continuous glucose monitoring. Created with BioRender.com.

We preregistered our study protocol at the Open Science Framework (https://osf.io/gpr52). The reported data (see Fig. 1 for a schematic overview of measures and main findings) are part of a larger study examining the potential link between metabolic states and reward learning, and the reported analyses were not preregistered. Data from the magnetic resonance imaging (MRI) sessions, the tasks during the sessions, and those that followed the EMA (Influenca^49^) was not analysed for our research question and will be reported separately.

### Participants

The sample size was determined to ensure sufficient statistical power for all planned analyses, including between-subject comparisons (e.g., men vs. women). For the primary within-subject analyses reported here, we expected small-to-moderate effects (*d*_z_∼0.40, where *d*_z_ is the standardised mean difference for within-subject designs). Since we expected to include at least 80 participants completing the entire study (including MRI visits and after quality control), the resulting statistical power was 0.94 (1-*β*, where *β* is the Type II error rate). A total of 97 participants were invited to participate. Participants were included in the analysis if we collected at least 20 EMA runs with concurrent CGM data. The threshold was defined a priori in line with typical recommendations^50^^,^^51^ on the minimum number of units to derive robust slope estimates. We excluded 7 participants due to extended malfunction of CGM (e.g., detached sensors or loss of transmission). This led to a sample of 90 participants (46 women, *M*_age_: 24.27 ± 3.57 years, range: 18–34, *M*_BMI_: 24.71 ± 4.09 kg/m^2^, range: 18–36.7). According to standardised screenings, all participants reported being physically and mentally healthy (except for a higher BMI) and reported no history of neurological, neurosurgical, or cardiological disease or treatment. Participants were not excluded based on blood parameters; instead, individual differences in metabolic indices (e.g., HOMA-IR) were characterised in the analyses (see Table 1). All participants were instructed to complete an online questionnaire during the study period, providing sociodemographic data including education (categorial scale: high school diploma, university of applied sciences entrance qualification, university degree, other school degree, unknown), student status (categorical scale: student/trainee, no longer student/trainee, unknown), income (categorical scale with predefined brackets: <€250 to €3000–<€4000, prefer not to answer). Furthermore, they completed the International Physical Activity Questionnaire (IPAQ),^52^ the Alcohol Use Disorders Identification Test (AUDIT),^53^ the Fagerström Test for Nicotine Dependence (FTND),^54^ and the Cannabis Use Disorders Identification Test (CUDIT).^55^ Participants received fixed compensation of 160€ or partial course credits and additionally performance-dependent wins from the tasks and EMA.

**Table 1:** Descriptive statistics of demographic, metabolic, and psychological variables.

Parameter	Value
Age (years)	24.28 ± 3.57 (range: 18.00–34.00)
Sex (n (%))	Male: 44 (49%) Female: 46 (51%)
BMI (kg/m^2^)	24.71 ± 4.09 (range: 18.00–36.70)
	Underweight (<18.5): 1 (1.1%)
	Healthy (18.5–24.9): 52 (57.8%)
	Overweight (25.0–29.9): 26 (28.9%)
	Obesity (≥30.0): 11 (12.2%)
Fasting glucose (mg/dL)	83.03 ± 7.88 (range: 65.00–111.00)
Mean CGM glucose level (mg/dL)	110.25 ± 6.46 (range: 95.61–135.79)
Fasting insulin (mU/l)	7.15 ± 3.77 (range: 2.16–19.44)
HOMA-IR (median)	1.61 ± 0.82 (range: 0.46–4.35)
	Not elevated HOMA-IR (<2.5) 78 (86.7%)
	Elevated HOMA-IR (≥2.5) 12 (13.3%)
TyG index (median)	4.37 ± 0.22 (range: 3.85–5.02)
AUDIT (sum)	5.34 ± 4.00 (range: 0.00–19.00)
FTND (sum)	0.04 ± 0.26 (range: 0.00–2.00)
CUDIT (sum)	0.88 ± 2.91 (range: 0.00–20.00)
IPAQ (sum)	3431.38 ± 2971.98 (range: 0.00–14,436.00)
Sleep duration (min)	488 ± 59 (range: 330–690)
Mood state	35.76 ± 25.62 (range: −72.65 to 97.02)
Metabolic state	−27.57 ± 18.39 (range: −69.17 to 15.43)
Student status (n (%))	Student/trainee: 61 (70%) No longer student/trainee: 26 (30%)
Education (n (%))	High school diploma: 50 (57%) University degree: 28 (32%) University of Applied Sciences Entrance Qualification (Fachhochschulreife): 5 (5.7%) Other: 4 (4.6%)

*Note*. Values are presented as Mean ± Standard Deviation (SD) (range: min–max) unless otherwise specified. Age is reported in years, body mass index (BMI) in kg/m^2^, fasting glucose levels in mg/dL, mean glucose levels in continuous glucose monitoring (CGM) in mg/dL, and fasting insulin levels in mU/l. The homeostatic model assessment of insulin resistance (HOMA-IR) as a unitless index and the triglyceride-glucose (TyG) index are shown as median ± SD. Questionnaire sum scores: the Alcohol Use Disorders Identification Test (AUDIT) ranges from 0 to 40, ≥8 indicate hazardous drinking, and ≥20 suggest possible dependence; the Fagerström Test for Nicotine Dependence (FTND) ranges from 0 to 10, with higher values reflecting greater nicotine dependence; the Cannabis Use Disorders Identification Test (CUDIT) ranges from 0 to 40, ≥8 indicates hazardous cannabis use, and ≥12 suggest probable cannabis dependence; the International Physical Activity Questionnaire (IPAQ): sum represents the total number of minutes a person spends in physical activity per week. Self-reported sleep duration is reported in minutes per night for the mean ± SD. Mood and metabolic states are reported as standardized scores (range: −100 to 100). Categorical variables (sex, student status, education) are presented as absolute numbers (n) with corresponding percentages (%).

### Ethics

All participants provided their informed written consent before the experiment. The ethics committee of the Faculty of Medicine at the University of Tübingen approved the experiment (585|2020BO), and all procedures were carried out in accordance with the Declaration of Helsinki.

### Experimental procedure

**Fig. 2:**
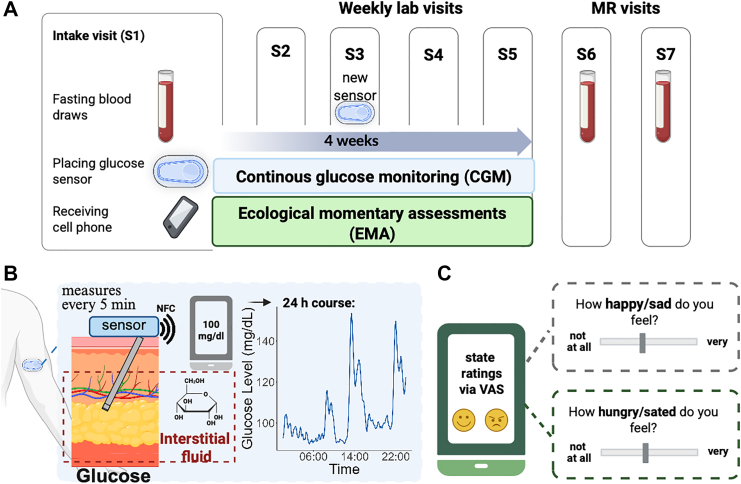
**Study description. A:** The schematic shows the experimental procedure, illustrating five weekly lab visits followed by 2 MR visits labeled from S1 to S7. After the first session (S1), participants undergo four weeks of continuous glucose monitoring (CGM) combined with ecological momentary assessment (EMA). **B:** The CGM system uses a small flex sensor placed at the posterior upper arm that is inserted in the subcutaneous fatty tissue. The glucose sensor, positioned beneath the skin surface layers (epidermis and dermis), measures glucose levels in the interstitial fluid within the subcutaneous adipose tissue. The data is wirelessly sent via near field communication (NFC) to the FreeStyle Libre 3 app on the participant's cell phone. The accuracy of the sensor concerning capillary glucose has been validated.^56^**C:** Participants rate their metabolic and mood states twice daily using a visual analog scale (VAS), ranging from 0 (not at all) to 100 (very). Created with BioRender.com.

Participants had five laboratory sessions over four weeks (Fig. 2), each occurring once a week, followed by two MRI sessions. In the first session (S1), they completed three tasks (Effort Allocation Task,^57^ Go/Nogo Reinforcement learning paradigm,^58^ Reward Ratings Task^59^), and state questions. Participants were fitted with a CGM sensor on the posterior upper arm during the first session, which continuously measured interstitial glucose throughout the day (FreeStyle Libre 3 sensors, Abbott GmbH, Abbott Diabetes Care, Wiesbaden, Germany). They received a cell phone to track their food intake, synchronise the data of the sensor via the FreeStyle Libre app, and play Influenca. EMA questions regarding mood and metabolic states were included in the gamified reinforcement learning task, ‘Influenca’, which they were asked to play twice a day over the following four weeks, with a minimum gap of 2 h between assessments.^49^ Before each run of ‘Influenca’, participants completed several questions capturing momentary metabolic (hunger, satiety, thirst, time since the last meal, and consumption of coffee or snacks in the previous 2 h), mental (mood including self-satisfaction, happiness, sadness, stress), and physical (physical health) states at that moment. Participants indicated their response on a visual analog scale, ranging from 0, representing “not at all”, to 100, representing “very much”. During the third session (i.e., after the second week), the first glucose sensor was replaced and then removed after two weeks. If a sensor fell off or was no longer reporting data, participants scheduled an appointment for a replacement. To determine fasting glucose, insulin, and triglyceride levels, we drew blood following a 12 h overnight fast (no caloric intake; water, tea, and coffee without milk or sugar with a negligible glucose effect^60^ permitted) at three visits.

### Data analysis

#### Preprocessing of the glucose data

**Fig. 3:**
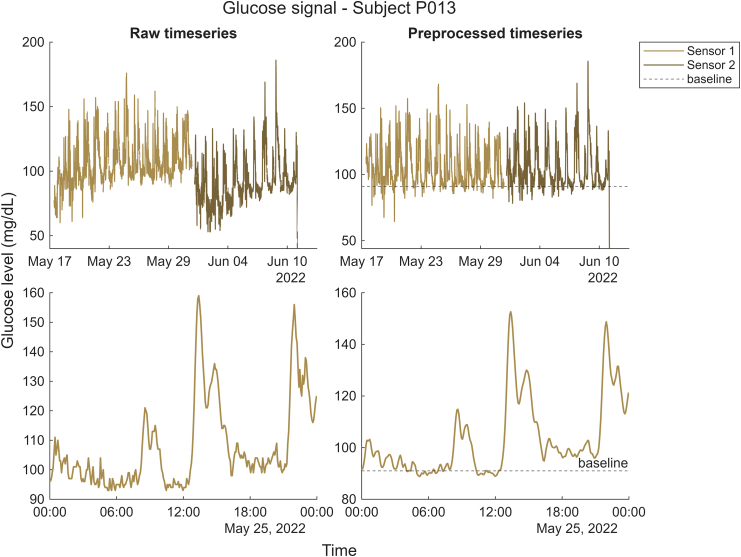
**Comparison of raw vs. preprocessed CGM data for one participant** (Top left) Raw time series with sensors/segments (represented with different colors) separated by > 20 min gaps. The non-linear drifts are visible for both sensors (Top right) Same glucose data after segment-wise non-physiological drift correction, addition of the participant's stable baseline (dashed line), and Gaussian smoothing. (Bottom) Daily glucose signal illustrating the reduction of noise after Gaussian smoothing. Data are summarized using a 5-min grid.

We extracted raw sensor glucose values and preprocessed the data according to well-established physiological signal correction steps to enhance data quality.^61^ To account for the variability between sensors (same model, at least two sensors to cover the four weeks) and missing data, we first segmented the data into sections marked by intervals of missing data longer than 20 min. Glucose levels during shorter data gaps (<20 min) were estimated via simple linear interpolation.^62^^,^^63^ The raw signals acquired by CGM sensors often exhibit non-physiological drift arising from calibration dynamics and sensor–tissue interactions.^64^ To remove the slow-varying drift while preserving the dynamics, we used MATLAB's ‘msbackadj’ function from the Bioinformatics toolbox, preferred over a single global linear detrend, which would fail to capture such nonlinearity while risking the distortion of shorter-timescale dynamics. To adjust each segment independently to correct for shifts, the ‘msbackadj’ function estimates a baseline for each segment and adjusts the segment. Next, we added a stable baseline value to these corrected glucose values for each participant. This enhances the interpretability of the glucose values and provides a common reference for the measurements from the two sensors used over the 4-week period. To establish this stable baseline for each participant, we identified the glucose period with the lowest glucose variability using the raw glucose values during the overnight fasting window (midnight to 8 am) since glycemic variability is typically lowest overnight for non-diabetic individuals.^65^, ^66^, ^67^ This was done using a sliding window of 1 h, advancing in steps of 5 min. The comparison between the raw and preprocessed glucose signal, including drift correction and baseline definition, is illustrated in the upper section of Fig. 3. Many CGM denoising approaches assume white Gaussian measurement noise.^68^, ^69^, ^70^ Consequently, after baseline correction, we implemented Gaussian smoothing with a window length of 5 data points (i.e., 25 min) to reduce noise while preserving the signal's essential characteristics (the smoothing effect can be observed in the lower section of Fig. 3). Subsequently, we partitioned the adjusted data into standardised 5-min intervals for further investigation. As the physiological delay of glucose transport from the vascular to the interstitial space is 5–6 min in healthy adults,^71^ we extracted the CGM value, which was recorded 5–10 min after the corresponding EMA. The MATLAB code is openly available on GitHub (https://github.com/neuromadlab/Glucose_Mood).

#### Behavioural data

To calculate a composite mood state, we used the ratings of the “happy” item and the “sad” items (range: from 0 to 100) by computing their difference, defined as “mood state” = (happy—sad), ranging from −100 (negative mood) to 100 (positive mood). Likewise, we calculated “metabolic state” = (hunger—satiety), ranging from −100 (satiety) to 100 (hungry). The calculation of these bipolar composite scores is consistent with validated dimensional approaches applied in EMA research.^72^ To explore the extent to which physiological changes in glucose levels are accurately perceived, we computed a novel measure of the interoceptive accuracy for each individual. This was done by extracting the individual estimates of the slope predicting the z-standardized metabolic state from a linear mixed-effects model (LME) of *z*-standardised glucose levels (slopes were inverted so that higher scores reflect better accuracy, similar to interoceptive coherence, Young et al., 2021^73^).

#### Insulin resistance derived from blood

As a validated and widely used measure of insulin resistance (i.e., interindividual differences in how well the body utilises glucose), we calculated the homeostasis model assessment of insulin resistance (HOMA-IR; higher HOMA-IR reflects lower insulin sensitivity) using the glucose and insulin levels measured from fasting blood samples.^74^ Most participants had three fasting blood samples to compute the median HOMA-IR (n = 1 had only two samples; n = 9 had only one). Since the distributions of HOMA-IR and glucose levels was skewed, we ln-transformed them for parametric analyses.^75^^,^^76^ To isolate insulin resistance from other correlated covariates, we residualised HOMA-IR values by adjusting for BMI, sex, and age.^77^^,^^78^

#### Statistics

We preprocessed CGM data with MATLAB vR2022b. All statistical analyses were conducted using R (v4.3.1, R Core Team, 2023). To partition the variance into between-person and within-person effects, we computed LMEs using lmerTest.^79^ The code of the statistical analysis and an overview of all model formulas are available on GitHub (https://github.com/neuromadlab/Glucose_Mood). To evaluate whether glucose levels alone are associated with mood, we modelled mood state as a function of glucose, followed by analysing happy and sad items separately to capture the differential glucose effect on positive and negative mood. Next, we investigated the indirect pathways of the mediation by examining how glucose levels influence hunger, satiety, and metabolic state ratings, and how metabolic state ratings affect mood states using LMEs. We investigated whether metabolic state ratings influence the association between glucose levels and mood, and whether insulin resistance serves as a moderator by including HOMA-IR values and their interaction with glucose and metabolic state. The model included z-standardised fixed effects for glucose levels, metabolic state ratings, and covariates (BMI, sex, residualised HOMA-IR, and age) to estimate average effects across the entire sample. To facilitate direct comparison across different scales and variables, all predictors (metabolic state, glucose levels, BMI, age, and HOMA-IR) were *z*-standardised across all observations and included as continuous variables. We included random intercepts and slopes for glucose levels and metabolic state to account for the inter-individual variance in repeated measurements (i.e., allowing individuals to deviate systematically from the group average). To examine whether metabolic state mediated the relationship between glucose levels and mood, we conducted a model-based mediation analysis using the R package “Mediation”^80^ with multilevel model input (10,000 resamples). This approach allows for a formal decomposition of total effects into direct and indirect components, while accounting for the nested data structure. To evaluate whether interoceptive accuracy (i.e., the correspondence of changes in glucose and hunger) is associated with mood state, we used linear models (LMs) to model average mood ratings (i.e., mean) and fluctuations in mood ratings (i.e., variability) as a function of interoceptive accuracy, including BMI, age, and sex as covariates (with interactions with interoceptive accuracy). We conducted sensitivity analyses excluding participants with obesity (BMI >30) or elevated HOMA-IR (>2.5), and evaluated additional covariates (menstrual cycle phase, assessment time, physical activity, smoking, alcohol use, sleep duration, and student status) using model comparisons. We considered α ≤ 0.05 as significant. Data was visualised using ggplot2,^81^ ggridges,^82^ ggpointdensity,^83^ and ggside.^84^

### Role of funders

The German Research Foundation (DFG) funded the study. The DFG reviewed and approved the study design for funding, but had no role in study design, data collection, analysis, interpretation, or writing of the manuscript. Authors retained full access to the data and final responsibility for publication.

## Results

**Fig. 4:**
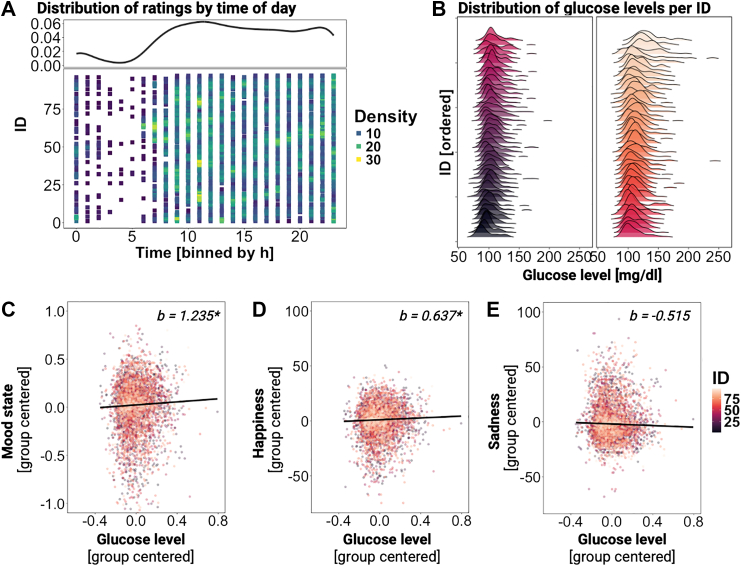
**Glucose levels are associated with mood. A:** The colour of each square represents the density of measurements for each participant across the day, binned by hours. The measurements are distributed and displayed in the average density curve during the hours of usual wake time. **B:** This density plot illustrates in two rows the distribution of glucose levels corresponding to the EMA for each participant, ordered by their median glucose levels. The lower panel displays the relationship between glucose levels and **C:** mood state, **D:** happiness, and **E:** sadness, indicating that when glucose levels are high, participants reported higher mood. Each dot represents an observation, with participants colour-coded. For visualisation, mood state, happiness, and sadness are group centered, and glucose levels are ln-transformed and group-centered to match the LME. Black lines indicate the relationship (fixed effect) across participants, indicating that with higher glucose levels, participants are happier. LME: ∗*p* < 0.05, ∗∗*p* < 0.01.

To investigate the association between glucose and metabolic state with mood (see Fig. S2 for the distribution), participants were asked to rate their states (Fig. 2C) while we recorded glucose levels using CGM (Fig. 2B). The analyses incorporated 4299 EMA runs from 90 participants. Most EMA (*M* = 47.76 ± 8.83 measurements per participant, range: 22–60) were recorded during the day (Fig. 4A; for demographics, see Table 1).

### Mood improves with higher glucose levels

To evaluate whether glucose levels are associated with mood, we initially examined the direct effect of glucose levels on mood as outcome with an LME (including BMI, age, sex, and HOMA-IR and their interactions with glucose levels to account for individual differences; *p*s > 0.05; LME assumptions are evaluated in Table S1 and Fig. S3 shows the distribution of modelled outcomes). Higher glucose levels were related to a better mood state (LME, *b* = 1.135, *t* (79) = 2.11, 95% CI [0.092, 2.165], *p* = 0.038, Fig. 4C; higher scores indicate more happiness vs. sadness). To explore whether there is a specific association with positive or negative mood, we estimated separate models and observed slightly stronger associations with happiness (LME, *b* = 0.637, *t* (76) = 2.38, 95% CI [0.118, 1.151], *p* = 0.020, Fig. 4D). In contrast, there was no significant association with sadness (LME, *b* = −0.515, *t* (77) = −1.43, 95% CI [−1.206, 0.183], *p* = 0.157, Fig. 4E). To examine potential non-linearity of the data, we used a generalized additive model (GAM) which confirmed that the relationship between glucose and mood is adequately described by a linear model (see Fig. S1).

### Hunger is associated with glucose levels and lower mood

**Fig. 5:**
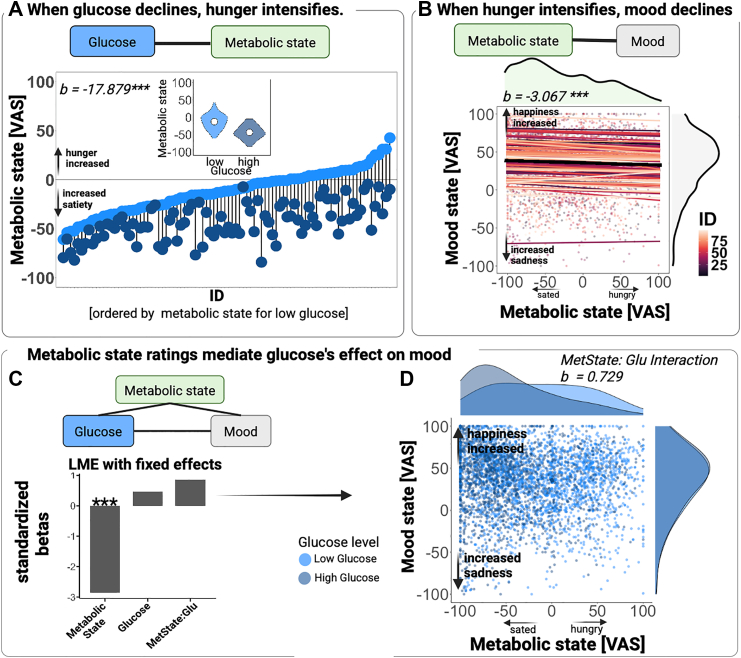
**Metabolic state and glucose levels, but not their interaction, are associated with lower mood. A:** The spaghetti plot displays the significant relationship between metabolic state ratings and mood state. Each dot depicts an observation with a color-coded grouping of participants. Colored lines show participant-specific estimates (random effect), while black indicates the average relationship (fixed effect) across participants. On top and to the right are the densities illustrated by the distribution of metabolic and mood states, respectively. **B:** Presents the metabolic states of each participant for the low and high glucose levels, which are ordered according to the participant's state score for their low glucose level. Dots represent the mean state values for the participant-specific low glucose (light blue) or high glucose (dark blue) level. To visualize differences in average metabolic state ratings between high and low glucose levels, participants were divided into two equally sized groups based on their group-centered glucose values (low vs. high glucose level). **C:** Standardized linear mixed-effects coefficients of metabolic state, ln-transformed glucose, and their interaction. **D:** The combination of scatter and density plots displays the interaction of metabolic state rating and glucose level on mood state, where the glucose levels are separated into low (light blue) and high (dark blue) glucose levels within each participant. LME: ∗*p* < 0.05, ∗∗*p* < 0.01. Created with BioRender.com.

To evaluate how strongly glucose levels are associated with hunger, we used an LME model with metabolic state ratings as the outcome and glucose levels as the predictor (LME model assumptions evaluated in Table S1). As expected, higher glucose levels were associated with less hunger (LME, *b* = −8.438, *t* (79) = −16.81, 95% CI [−9.395, −7.461], *p* < 0.001) and more satiety (LME, *b* = 9.678, *t* (80) = 16.48, 95% CI [8.349, 10.572], *p* < 0.001), leading to a strong association with the metabolic state (Fig. 5A; hungry—sated, LME, *b* = −17.879, *t* (75) = −17.49, 95% CI [−19.825, −15.886], *p* < 0.001; Table S2 for full results including wild bootstrap CIs). To consider inter-individual differences, the extended model also included BMI, age, sex, and HOMA-IR, and their interactions with glucose levels. Males had a higher metabolic state compared to females (LME, Sex: *b* = 14.191, *t* (85) = 3.65, 95% CI [6.710, 21.799], *p* < 0.001). However, none of the covariates showed a significant interaction with glucose levels (*p*s > 0.05).

To evaluate whether hunger and mood are associated, we analysed the effect of metabolic state on mood (higher scores indicate more happiness vs. sadness) using an LME that included only metabolic state as the predictor. As expected, when participants were hungrier, they also reported lower mood (LME, *b* = −3.067, *t* (88) = −4.84, 95% CI [−4.289, −1.841], *p* < 0.001, Fig. 5B). To consider inter-individual differences, an extended model also included BMI, age, sex, and adjusted values of HOMA-IR. None of the covariates significantly affected mood state ratings (*p*s > 0.05). To sum up, these findings indicate that glucose levels are a physiological signature of metabolic state, and both glucose levels and metabolic state are associated with mood.

### Metabolic state ratings mediate the effect of glucose on mood

To investigate if ratings of metabolic state mediate the association between glucose levels and mood, we first used an LME, including metabolic state, glucose level, and their interaction as predictors (Fig. 5C). Glucose levels alone were no longer significantly related to mood (LME, *b* = 0.356, *t* (80) = 0.56, 95% CI [−0.890, 1.560], *p* = 0.577) when self-reported metabolic state was also included in the LME. However, the effect of metabolic state ratings on mood (i.e., worse mood when hungrier, LME, *b* = −2.953, *t* (95) = −4.31, 95% CI [−4.298, −1.633], *p* < 0.001) was still significant independent of glucose levels. The interaction between glucose levels and metabolic state did not significantly influence mood (LME, *b* = 0.729, *t* (108) = 1.18, 95% CI [−0.481, 1.913], *p* = .241, Fig. 5D). Moreover, we observed a significant interaction of Sex × Metabolic state × Glucose levels (LME, *b* = 2.678 *t* (68) = 2.23, 95% CI [0.389, 4.984], *p* = 0.043), suggesting that the association was stronger in females (for details, see the following results on interoceptive accuracy).

We then conducted a mediation analysis for LMEs (R package *mediation*). In line with the LME results, the estimated indirect effect of glucose via metabolic state was significant (LME-based, average causal mediation effect (ACME) = 0.775, 95% CI [0.331, 1.224], *p* < 0.001). In contrast, the direct effect of glucose on mood was not significant (LME-based, average direct effect (ADE) = 0.333, 95% CI [−0.747, 1.428], *p* = 0.544). The estimated proportion mediated (ratio of ACME to total effect) was 0.680 (LME-based, 95% CI [0.172, 3.941], *p* = 0.039), suggesting that much of the observed association is explained by metabolic state ratings. However, the wide confidence intervals for the proportion mediated and the total effect (LME-based, 1.108, 95% CI [0.056, 2.158], *p* = 0.039) indicate that the overall impact of glucose on mood was small and variable. This pattern is consistent with an indirect-only mediation.

To examine whether insulin resistance modulates the link between glucose, metabolic state, and mood, we included the adjusted values of HOMA-IR and their interactions with glucose levels and metabolic state ratings, sex, age, and BMI. We found no significant interaction between HOMA-IR, glucose levels, and metabolic state on mood (LME, *b* = 0.777, *t* (122) = 1.25, 95% CI [−0.433, 1.996], *p* = 0.215), and no significant main effect (*p*s > 0.05). Taken together, our findings suggest that a conscious self-reported metabolic state mediates the association between glucose and mood.

### Interoceptive accuracy is associated with fluctuations in mood

**Fig. 6:**
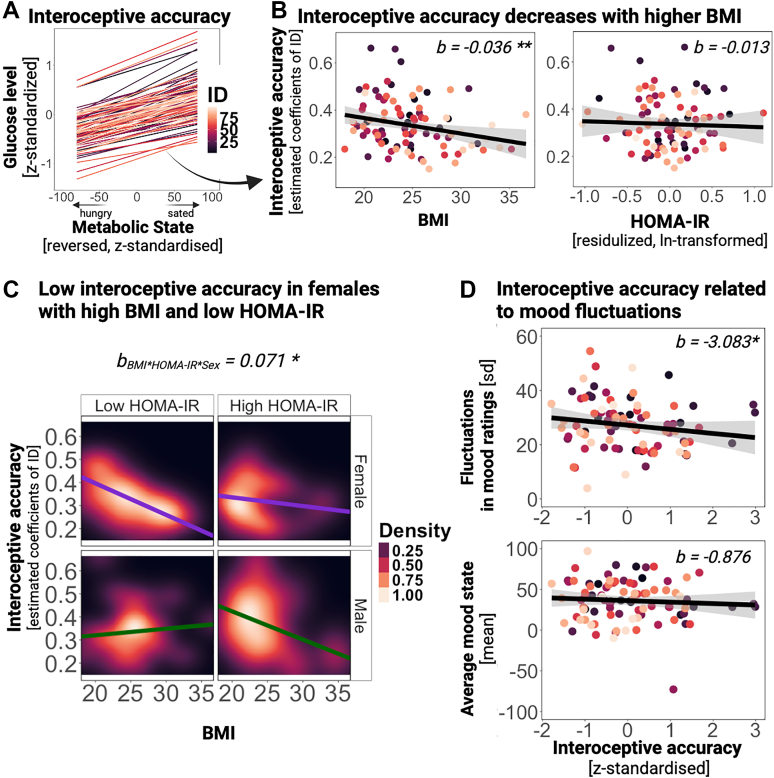
**Effect of metabolic factors (BMI, HOMA-IR) on interoceptive accuracy and its association with fluctuations in mood. A:** Depicted are the individual standardized slopes (i.e., interoceptive accuracy) as individual lines derived from an LME, where the differences in glucose levels (ln-transformed, *z*-standardized) were predicted with the metabolic state ratings (reversed, *z*-standardized). **B:** Interoceptive accuracy is associated with BMI, but not with ln-transformed and residualised HOMA-IR. Each dot depicts an observation with a color-coded grouping of participants. The regression lines were computed via a robust linear regression method and are displayed with 95% confidence intervals. **C:** Interoceptive accuracy is associated with BMI more strongly in females with lower HOMA-IR, indicating an interplay of metabolic health and sex on interoceptive accuracy. The plot shows the density distribution of interoceptive accuracy (on the y-axis) against BMI (on the x-axis), which is stratified across two levels of HOMA-IR by splitting participants into two equally sized groups based on their residualised HOMA-IR (low vs. high HOMA-IR. Each represents a distinct subgroup within the study population. The regression lines were computed via a robust linear regression method and are displayed with 95% confidence intervals. The color gradient of the background density ranges from black, indicating low density, to white, which indicates a high density of observations within the BMI and interoceptive accuracy. The background color gradient depicts the two-dimensional kernel density of observations to illustrate their distribution (black = low density; white = high density). **D:** Interoceptive accuracy (*z*-standardized, grand mean centered) is associated with fluctuations in mood ratings (standard deviation within a person) and the average mood state (mean rating per participant).

Since the relationship between glucose and mood is mediated by self-reported metabolic state (i.e., interoception), we explored whether interoceptive accuracy explained associations with average mood ratings (i.e., mean) and fluctuations in mood ratings (i.e., variability). First, interoceptive accuracy was computed by extracting the individual estimates from an LME, where differences in *z*-standardised glucose levels were predicted by the *z*-standardized metabolic state ratings (Fig. 6A), and inverting the estimates so that higher values reflect better accuracy. To investigate potential factors influencing the predictor of interoceptive accuracy, we examined the association between interoceptive accuracy and BMI, HOMA-IR, sex, and age. As expected, interoceptive accuracy was lower with a higher BMI (LM, *b* = −0.036, *t* (74) = −2.88, 95% CI [−0.061, −0.011], *p* = 0.005, Fig. 6C), but there were no associations with HOMA-IR alone (*b* = −0.013, *t* (74) = −1.02, 95% CI [−0.037, 0.012], *p* = 0.312, Fig. 6B), sex (*b* = 0.038, *t* (74) = 1.62, 95% CI [−0.009, 0.084], *p* = 0.110) or age (*b* = 0.0003, *t* (74) = 0.02, 95% CI [−0.025, 0.026], *p* = 0.984). However, there was a significant interaction between HOMA-IR (adjusted for BMI, age, and sex), BMI, and sex (*b* = 0.071, *t* (74) = 2.39, 95% CI [0.012, 0.131], *p* = 0.019, Fig. 6D), indicating that the interoceptive accuracy was reduced for females with high BMI and low HOMA-IR and for males with high HOMA-IR. When examining mood, we found that interoceptive accuracy was not associated with average mood ratings (i.e., mean, LM, *b* = −0.876, *t* (82) = −0.27, 95% CI [−7.246, 5.495], *p* = 0.785, Fig. 4B). Instead, interoceptive accuracy was associated with fluctuations in mood ratings (i.e., variability, LM, *b* = −3.083, *t* (82) = −2.92, 95% CI [−5.187, −0.979], *p* = 0.005, Fig. 4B). Variability increased with higher BMI (*b* = −2.602, t (82) = −2.48, 95% CI [−4.693, −0.510], *p* = 0.015), and the effect of interoceptive accuracy differed by sex (*b* = 5.691, t (82) = 2.84, 95% CI [1.711, 9.670], *p* = 0.006). To sum up, interoceptive accuracy seems to play a crucial role in fluctuations in mood rather than average mood state.

### Robustness and sensitivity analyses with additional covariates

To test the robustness of our findings, we performed sensitivity analyses excluding either 11 individuals with obesity (BMI ≥30 kg/m^2^) or 12 individuals with elevated HOMA-IR (≥2.5), where 8 individuals had both a BMI ≥30 kg/m^2^ and an HOMA-IR (≥2.5). Although the direct effect of glucose on mood was more variable after the exclusions, the results were largely consistent across these analyses, showing comparable associations in these subgroups (see Tables S3 and S4 and Fig. S4), and our conclusions were unchanged. We then evaluated the effect of additional covariates (menstrual cycle phase (see Fig. S5 and Tables S5 and S6), assessment time, physical activity, smoking behaviour, alcohol use, self-reported sleep duration (see Table S7), and student status) using model comparisons (see Table S8). Of all additional covariates, only assessment time improved model fit. Including assessment time as a linear predictor (for the temporal trend, see Fig. S6) led to small yet significant interactions (i.e., as a fixed effect with Glucose level × Metabolic State × Time; see Supplementary Material I), but did not alter the previously reported conclusions. Hence, assessment time is not driving these associations but may account for some of the observed variability in the estimates.

## Discussion

Hunger worsens mood, but whether this is due to consciously sensed hunger or subconscious changes in glucose levels remains unclear. Using a deep phenotyping approach in naturalistic settings, we found an association between glucose and mood that was attenuated when metabolic state ratings were considered. This suggests that self-reported metabolic state mediates glucose's effects on mood, refuting the idea of a largely subconscious influence. In support of an allostatic process subserving energy and mood regulation, we found that associations with positive mood were more pronounced compared to negative mood and the linear association speaks against a restricted negative valence signal as posited by homeostatic theories. In other words, it is conceivable that postprandial states are associated with positive valence beyond the simple homeostatic relief of hunger in metabolically healthy participants (as suggested by associations with fasting levels of ghrelin^77^), whereas negative valence-related signals may play a larger role in individuals with metabolic dysfunctions. We conclude that hunger-induced mood changes reflect conscious sensing of metabolic state, with hunger acting as an early metabolic signal that triggers a behavioural and affective cascade to tune adaptive behaviour.^5^

Consistent with prior research, we observed a strong association between hunger and lower mood in healthy adults.^1^^,^^36^^,^^37^ While traditionally seen as a drive to reduce energy deficits,^31^ hunger is now recognised as intertwined with mood.^77^ Hunger ratings reflect multidimensional interoceptive sensing, integrating bodily cues (e.g., rumbling stomach, feeling cold, fatigue, irritability).^35^^,^^85^^,^^86^ People process interoceptive signals across focal bodily sensations (e.g., empty stomach), diffuse sensations (e.g., nausea), and affective states (irritability, boredom).^86^^,^^87^ Thus, glucose levels correlated robustly with metabolic state ratings, in line with prior research.^19^^,^^88^, ^89^, ^90^ This aligns with the psychological construction of emotion theory, which posits emotions emerge from interpreting internal states and external cues.^40^ Accordingly, homeostatic processes (e.g., hunger signalling energy deficit) generate core affects that shape generalised mood states and the basis of emotions.^43^ This effect is pronounced when individuals lack awareness of their bodily needs.^1^^,^^39^ However, metabolic state ratings mediated the association between mood and glucose, indicating that conscious sensing of metabolic states affects mood, not glucose per se. This aligns with meta-analytic evidence refuting subconscious links between mood, self-control, and glucose depletion^91^, ^92^, ^93^, ^94^ and challenging broad claims about direct links with self-control.^95^^,^^96^ Our findings remained unchanged when adjusting for covariates (menstrual cycle phase, assessment time, physical activity, smoking behaviour, alcohol use, sleep duration, and student status); only the assessment time showed a small interaction with glucose and metabolic state, but this did not alter the conclusions. Taken together, our findings support the psychological construction of emotion theory by demonstrating that hunger contributes to mood decline via conscious sensing.

Interoception plays a crucial role in mood regulation and well-being.^97^, ^98^, ^99^ Our findings show that higher interoceptive accuracy of metabolic state is associated with fewer mood fluctuations but not average mood states. These results align well with the theory that interoception is an important aspect of maintaining mental health. Mood variability and disrupted integration of bodily signals may contribute mechanistically to mental disorders, specifically mood disorders.^47^^,^^100^ Our observations partially align with Young et al., 2019,^101^ who showed that individuals with higher interoceptive accuracy had reduced hunger and anxiety after glucose intake. Neither Young et al.^101^ nor Zamariola et al.^102^ found a link between interoceptive accuracy (via heartbeat counting task) and mood, supporting our finding that interoceptive accuracy is not associated with average mood state. While most studies focus on cardiac interoception,^97^^,^^103^ ours is the first comprehensive approach to examine metabolic interoception in detecting glucose changes using combined EMA and CGM. Crucially, recent experimental work using rigorous psychophysical testing in large healthy cohorts has demonstrated that interoceptive accuracy is not a domain-general phenomenon^104^ (preprint, version posted Aug. 2025), contradicting popular ideas that cardiac interoception can serve as a general proxy for interoception more broadly. However, interoceptive deficits are linked to higher BMI,^105^ highlighting a connection between metabolic sensing and weight regulation^106^^,^^107^ and pointing to a potential sex-specific risk factor for future studies in women, who have a higher prevalence of (comorbid) eating and mood disorders.^108^ In contrast, glucose metabolism did not moderate mood regulation in metabolically healthy individuals, as they were unrelated to insulin sensitivity or BMI. We conclude that interoceptive accuracy helps regulate mood by enhancing responsiveness to metabolic signals.^109^

Despite showing that metabolic state ratings account for mood-related effects of hunger, limitations remain. First, we focus on glucose as one signal related to energy metabolism, while additional endocrine signals, such as ghrelin,^77^ continuous monitoring of cortisol,^110^ or sex hormones,^111^^,^^112^ also play a role. Although we observed sex differences, additional control analyses did not substantiate that these differences are driven by cycle-related variation (see Fig. S5 and Tables S5 and S6). Future studies specifically addressing changes in insulin sensitivity across the menstrual cycle^113^^,^^114^ could provide additional insights. Second, although metabolic state ratings explained a significant portion of the association between glucose and mood, the remaining proportion may reflect other mediators (e.g., circadian phase, food intake differing in macronutrients) or even random variation. More generally, mood integrates diverse bodily signals, and it remains to be tested whether hunger uniquely drives mood changes compared to other homeostatic challenges, such as pain or temperature. Third, despite the known limitations of CGM in healthy participants,^115^ for example, to evaluate specific meals, our study used several steps, such as re-referencing to a participant-specific stable fasting baseline (defined as the minimum over days in the lowest-variability overnight window), which markedly improved signal quality and indicated robust associations with subjective ratings of metabolic states. Fourth, while we found an association between glucose levels and mood in healthy participants, it remains unclear whether such associations would occur in individuals with metabolic or mental disorders.^116^, ^117^, ^118^ Future research in clinical samples will help clarify the potential role of interoception in aberrant mood regulation.

To summarise, metabolic state ratings mediate the link between glucose levels and mood. Using a highly powered deep phenotyping design with repeated CGM and self-reported metabolic and mood measures in naturalistic settings, we further show that higher interoceptive accuracy is associated with lower mood fluctuations, highlighting the role of metabolic interoception in mood regulation. Our findings suggest that conscious signals of the body's metabolic state affect mood in mostly metabolically healthy participants, aligning with theories where changes in mood drive behavioural adjustments. This advances our understanding of mood regulation and supports the psychological construction of emotion theory, emphasising interoception's role in maintaining allostasis.

## Contributors

NBK was responsible for the study concept and design. MK, MG, KK, & AK collected data under supervision by NBK and BD. AK, MG, BD, & NBK conceived the method, and MBT and AK preprocessed the data. MK, KK, & NBK performed the data analysis. Every author had full access to the dataset, and AK verified the underlying data and independently reproduced key analyses. KK, MK, & NBK wrote the manuscript. All authors contributed to the interpretation of findings, provided critical revision of the manuscript for important intellectual content, and approved the final version for publication.

## Data sharing statement

Anonymized study data, comprising the preprocessed variables of the main analyses (e.g., ratings of mood and metabolic state, momentary CGM glucose at the time of rating, interoceptive accuracy, BMI, age, HOMA-IR, and sex), are openly available. The data and code for preprocessing the glucose data and performing the main analyses are openly available on GitHub https://github.com/neuromadlab/Glucose_Mood.

## Declaration of interests

The authors declare no competing financial interests.

## References

[1] MacCormack J.K., Lindquist K.A. (2019). Feeling hangry? When hunger is conceptualized as emotion. Emotion.

[2] Piccolo M., Milos G., Bluemel S. (2020). Effects of hunger on mood and affect reactivity to monetary reward in women with obesity – a pilot study. PLoS One.

[3] Bennett D., Davidson G., Niv Y. (2022). A model of mood as integrated advantage. Psychol Rev.

[4] Eldar E., Rutledge R.B., Dolan R.J., Niv Y. (2016). Mood as representation of momentum. Trends Cogn Sci.

[5] Teckentrup V., Kroemer N.B. (2024). Mechanisms for survival: vagal control of goal-directed behavior. Trends Cogn Sci.

[6] Lyra E Silva N.D.M., Lam M.P., Soares C.N. (2019). Insulin resistance as a shared pathogenic mechanism between depression and type 2 diabetes. Front Psychiatry.

[7] Vancampfort D., Stubbs B., Mitchell A.J. (2015). Risk of metabolic syndrome and its components in people with schizophrenia and related psychotic disorders, bipolar disorder and major depressive disorder: a systematic review and meta-analysis. World Psychiatry.

[8] Dienel G.A. (2019). Brain glucose metabolism: integration of energetics with function. Physiol Rev.

[9] Mergenthaler P., Lindauer U., Dienel G.A., Meisel A. (2013). Sugar for the brain: the role of glucose in physiological and pathological brain function. Trends Neurosci.

[10] Gold A.E., MacLeod K.M., Frier B.M., Deary I.J. (1995). Changes in mood during acute hypoglycemia in healthy participants. J Pers Soc Psychol.

[11] Owen L., Scholey A.B., Finnegan Y., Hu H., Sünram-Lea S.I. (2012). The effect of glucose dose and fasting interval on cognitive function: a double-blind, placebo-controlled, six-way crossover study. Psychopharmacology (Berl).

[12] Owens D., Parker P.Y., Benton D. (1997). Blood glucose and subjective energy following cognitive demand. Physiol Behav.

[13] Bushman B.J., DeWall C.N., Pond R.S., Hanus M.D. (2014). Low glucose relates to greater aggression in married couples. Proc Natl Acad Sci.

[14] Denson T.F., von Hippel W., Kemp R.I., Teo L.S. (2010). Glucose consumption decreases impulsive aggression in response to provocation in aggressive individuals. J Exp Soc Psychol.

[15] Benton D., Owens D. (1993). Is raised blood glucose associated with the relief of tension?. J Psychosom Res.

[16] Campfield L.A., Brandon P., Smith F.J. (1985). On-line continuous measurement of blood glucose and meal pattern in free-feeding rats: the role of glucose in meal initiation. Brain Res Bull.

[17] Campfield L.A., Smith F.J. (1986). Functional coupling between transient declines in blood glucose and feeding behavior: temporal relationships. Brain Res Bull.

[18] Melanson K.J., Westerterp-Plantenga M.S., Saris W.H.M., Smith F.J., Campfield L.A. (1999). Blood glucose patterns and appetite in time-blinded humans: carbohydrate versus fat. Am J Physiol.

[19] Schultes B., Oltmanns K.M., Kern W., Fehm H.L., Born J., Peters A. (2003). Modulation of hunger by plasma glucose and metformin. J Clin Endocrinol Metab.

[20] Woods S.C., Lutz T.A., Geary N., Langhans W. (2006). Pancreatic signals controlling food intake; insulin, glucagon and amylin. Philos Trans R Soc B Biol Sci.

[21] Van De Rest O., Van Der Zwaluw N.L., De Groot L.C.P.G.M. (2018). Effects of glucose and sucrose on mood: a systematic review of interventional studies. Nutr Rev.

[22] Green M.W., Taylor M.A., Elliman N.A., Rhodes O. (2001). Placebo expectancy effects in the relationship between glucose and cognition. Br J Nutr.

[23] Markus C.R. (2007). Effects of carbohydrates on brain tryptophan availability and stress performance. Biol Psychol.

[24] Scholey A., Savage K., O'Neill B.V. (2014). Effects of two doses of glucose and a caffeine–glucose combination on cognitive performance and mood during multi-tasking. Human Psychopharmacology.

[25] Seo Y., Peacock C.A., Gunstad J., Burns K.J., Pollock B.S., Glickman E.L. (2014). Do glucose containing beverages play a role in thermoregulation, thermal sensation, and mood state?. J Int Soc Sports Nutr.

[26] Ullrich S., De Vries Y.C., Kühn S., Repantis D., Dresler M., Ohla K. (2015). Feeling smart: effects of caffeine and glucose on cognition, mood and self-judgment. Physiol Behav.

[27] Keshet A., Shilo S., Godneva A. (2023). CGMap: characterizing continuous glucose monitor data in thousands of non-diabetic individuals. Cell Metab.

[28] Shilo S., Keshet A., Rossman H. (2024). Continuous glucose monitoring and intrapersonal variability in fasting glucose. Nat Med.

[29] Wagner J., Armeli S., Tennen H., Bermudez-Millan A., Wolpert H., Pérez-Escamilla R. (2017). Mean levels and variability in affect, diabetes self-care behaviors, and continuously monitored glucose: a daily study of latinos with type 2 diabetes. Psychosom Med.

[30] Zink J., Nicolo M., Imm K. (2020). Interstitial glucose and subsequent affective and physical feeling states: a pilot study combining continuous glucose monitoring and ecological momentary assessment in adolescents. J Psychosom Res.

[31] Aunger R., Curtis V. (2013). The anatomy of motivation: an evolutionary-ecological approach. Biol Theory.

[32] Beaulieu K., Blundell J. (2021). The psychobiology of hunger – a scientific perspective. Topoi.

[33] Betley J.N., Xu S., Cao Z.F.H. (2015). Neurons for hunger and thirst transmit a negative-valence teaching signal. Nature.

[34] Reichenbach A., Clarke R.E., Stark R. (2022). Metabolic sensing in AgRP neurons integrates homeostatic state with dopamine signalling in the striatum. eLife.

[35] Monello L.F., Mayer J. (1967). Hunger and satiety sensations in men, women, boys, and girls. Am J Clin Nutr.

[36] Swami V., Hochstöger S., Kargl E., Stieger S. (2022). Hangry in the field: an experience sampling study on the impact of hunger on anger, irritability, and affect. PLoS One.

[37] De Rivaz R., Swendsen J., Berthoz S., Husky M., Merikangas K., Marques-Vidal P. (2022). Associations between hunger and psychological outcomes: a large-scale ecological momentary assessment study. Nutrients.

[38] Nettle D. (2017). Does hunger contribute to socioeconomic gradients in behavior?. Front Psychol.

[39] Ackermans M.A., Jonker N.C., Bennik E.C., De Jong P.J. (2022). Hunger increases negative and decreases positive emotions in women with a healthy weight. Appetite.

[40] Barrett L.F. (2006). Solving the emotion paradox: categorization and the experience of emotion. Pers Soc Psychol Rev.

[41] Barrett L.F. (2017). The theory of constructed emotion: an active inference account of interoception and categorization. Soc Cogn Affect Neurosci.

[42] MacCormack J.K., Lindquist K.A. (2017). Bodily contributions to emotion: Schachter's legacy for a psychological constructionist view on emotion. Emot Rev.

[43] Russell J.A. (2003). Core affect and the psychological construction of emotion. Psychol Rev.

[44] Craig A.D. (2002). How do you feel? Interoception: the sense of the physiological condition of the body. Nat Rev Neurosci.

[45] Feldman M.J., Bliss-Moreau E., Lindquist K.A. (2024). The neurobiology of interoception and affect. Trends Cogn Sci.

[46] Feldman M.J., Ma R., Lindquist K.A., Murphy J., Brewer R. (2024). Interoception.

[47] Barrett L.F., Simmons W.K. (2015). Interoceptive predictions in the brain. Nat Rev Neurosci.

[48] Simmons W.K., DeVille D.C. (2017). Interoceptive contributions to healthy eating and obesity. Curr Opin Psychol.

[49] Neuser M.P., Kühnel A., Kräutlein F., Teckentrup V., Svaldi J., Kroemer N.B. (2023). Reliability of gamified reinforcement learning in densely sampled longitudinal assessments. PLOS Digit Health.

[50] Brysbaert M., Stevens M. (2018). Power analysis and effect size in mixed effects models: a tutorial. J Cogn.

[51] Weichenthal S., Baumgartner J., Hanley J.A. (2017). Sample size estimation for random-effects models. Epidemiology.

[52] Craig C.L., Marshall A.L., Ainsworth B.E. (2003). International physical activity questionnaire: 12-Country reliability and validity. Med Sci Sports Exerc.

[53] Saunders J.B., Aasland O.G., Babor T.F., De La Fuente J.R., Grant M. (1993). Development of the Alcohol Use Disorders Identification Test (AUDIT): WHO collaborative project on early detection of persons with harmful alcohol consumption-II. Addiction.

[54] Fagerstrom K.O., Schneider N.G. (1989). Measuring nicotine dependence: a review of the fagerstrom tolerance questionnaire. J Behav Med.

[55] Adamson S.J., Sellman J.D. (2003). A prototype screening instrument for cannabis use disorder: the Cannabis Use Disorders Identification Test (CUDIT) in an alcohol-dependent clinical sample. Drug Alcohol Rev.

[56] Alva S., Bailey T., Brazg R. (2022). Accuracy of a 14-day factory-calibrated continuous glucose monitoring system with advanced Algorithm in pediatric and adult population with diabetes. J Diabetes Sci Technol.

[57] Neuser M.P., Teckentrup V., Kühnel A., Hallschmid M., Walter M., Kroemer N.B. (2020). Vagus nerve stimulation boosts the drive to work for rewards. Nat Commun.

[58] Kühnel A., Teckentrup V., Neuser M.P. (2020). Stimulation of the vagus nerve reduces learning in a go/no-go reinforcement learning task. Eur Neuropsychopharmacol.

[59] Müller F.K., Teckentrup V., Kühnel A., Ferstl M., Kroemer N.B. (2022). Acute vagus nerve stimulation does not affect liking or wanting ratings of food in healthy participants. Appetite.

[60] Sciarrillo C.M., Keirns B.H., Elliott D.C., Emerson S.R. (2021). The effect of black coffee on fasting metabolic markers and an abbreviated fat tolerance test. Clin Nutr ESPEN.

[61] Visser M.M., Gillard P. (2024). Best practices in collecting and reporting continuous glucose monitoring data in research settings. Nat Metab.

[62] Fonda S.J., Lewis D.G., Vigersky R.A. (2013). Minding the gaps in continuous glucose monitoring: a method to repair gaps to achieve more accurate glucometrics. J Diabetes Sci Technol.

[63] Stewart K.W., Pretty C.G., Shaw G.M., Chase J.G. (2018). Interpretation of retrospective BG measurements. J Diabetes Sci Technol.

[64] Acciaroli G., Vettoretti M., Facchinetti A., Sparacino G. (2018). Calibration of minimally invasive continuous glucose monitoring sensors: state-of-the-art and current perspectives. Biosensors.

[65] Shah V.N., DuBose S.N., Li Z. (2019). Continuous glucose monitoring profiles in healthy nondiabetic participants: a multicenter prospective study. J Clin Endocrinol Metab.

[66] Liang Z. (2022). Mining associations between glycemic variability in awake-time and in-sleep among non-diabetic adults. Front Med Technol.

[67] Sofizadeh S., Pehrsson A., Ólafsdóttir A.F., Lind M. (2022). Evaluation of reference metrics for continuous glucose monitoring in persons without diabetes and prediabetes. J Diabetes Sci Technol.

[68] Zhao H., Zhao C., Gao F. (2018). An automatic glucose monitoring signal denoising method with noise level estimation and responsive filter updating. Biomed Signal Process Control.

[69] Yadav J., Srivastav N., Agarwal S., Rani A., Pant M., Sharma T.K., Verma O.P., Singla R., Sikander A. (2020). Soft computing: theories and applications.

[70] Camerlingo N., Siviero I., Vettoretti M., Sparacino G., Del Favero S., Facchinetti A. (2023). Bayesian denoising algorithm dealing with colored, non-stationary noise in continuous glucose monitoring timeseries. Front Bioeng Biotechnol.

[71] Basu A., Dube S., Slama M. (2013). Time lag of glucose from intravascular to interstitial compartment in humans. Diabetes.

[72] Russell J.A. (1980). A circumplex model of affect. J Pers Soc Psychol.

[73] Young H.A., Gaylor C.M., de-Kerckhove D., Benton D. (2021). Individual differences in sensory and expectation driven interoceptive processes: a novel paradigm with implications for alexithymia, disordered eating and obesity. Sci Rep.

[74] Matthews D.R., Hosker J.P., Rudenski A.S., Naylor B.A., Treacher D.F., Turner R.C. (1985). Homeostasis model assessment: insulin resistance and ?-cell function from fasting plasma glucose and insulin concentrations in man. Diabetologia.

[75] Emoto M., Nishizawa Y., Maekawa K. (1999). Homeostasis model assessment as a clinical index of insulin resistance in type 2 diabetic patients treated with sulfonylureas. Diabetes Care.

[76] Kroemer N.B., Krebs L., Kobiella A. (2013). Fasting levels of ghrelin covary with the brain response to food pictures: ghrelin and food-cue reactivity. Addict Biol.

[77] Fahed R., Schulz C., Klaus J., Ellinger S., Walter M., Kroemer N.B. (2023). Ghrelin is associated with an elevated mood after an overnight fast in depression. http://medrxiv.org/lookup/doi/10.1101/2023.12.18.23300133.

[78] Schulz C., Klaus J., Peglow F. (2024). Blunted anticipation but not consummation of food rewards in depression. http://medrxiv.org/lookup/doi/10.1101/2024.03.26.24304849.

[79] Kuznetsova A., Brockhoff P.B., Christensen R.H.B. (2017). lmerTest package: tests in linear mixed effects models. J Stat Softw.

[80] Tingley D., Yamamoto T., Hirose K., Keele L., Imai K. (2014). mediation : R package for causal mediation analysis. J Stat Softw.

[81] Wickham H. (2016).

[82] Wilke C.O. (2017). ggridges: ridgeline plots in ‘ggplot2’. https://CRAN.R-project.org/package=ggridges.

[83] Kremer L.P.M. (2019). ggpointdensity: a cross between a 2D density plot and a scatter plot. https://CRAN.R-project.org/package=ggpointdensity.

[84] Landis J. (2021).

[85] Hams A., Wardle J. (1987). The feeling of hunger. Br J Clin Psychol.

[86] Stevenson R.J., Hill B.J., Hughes A. (2023). Interoceptive hunger, eating attitudes and beliefs. Front Psychol.

[87] Stevenson R.J., Mahmut M., Rooney K. (2015). Individual differences in the interoceptive states of hunger, fullness and thirst. Appetite.

[88] Campfield L.A., Smith F.J., Rosenbaum M., Hirsch J. (1996). Human eating: evidence for a physiological basis using a modified paradigm. Neurosci Biobehav Rev.

[89] Lemmens S.G., Martens E.A., Kester A.D., Westerterp-Plantenga M.S. (2011). Changes in gut hormone and glucose concentrations in relation to hunger and fullness. Am J Clin Nutr.

[90] Nederkoorn C., Guerrieri R., Havermans R.C., Roefs A., Jansen A. (2009). The interactive effect of hunger and impulsivity on food intake and purchase in a virtual supermarket. Int J Obes.

[91] Dang J. (2016). Testing the role of glucose in self-control: a meta-analysis. Appetite.

[92] Kurzban R. (2010). Does the brain consume additional glucose during self-control tasks?. Evol Psychol.

[93] Orquin J.L., Kurzban R. (2016). A meta-analysis of blood glucose effects on human decision making. Psychol Bull.

[94] Raichle M.E., Mintun M.A. (2006). Brain work and brain imaging. Annu Rev Neurosci.

[95] Gailliot M.T., Baumeister R.F., DeWall C.N. (2007). Self-control relies on glucose as a limited energy source: willpower is more than a metaphor. J Pers Soc Psychol.

[96] Penckofer S., Quinn L., Byrn M., Ferrans C., Miller M., Strange P. (2012). Does glycemic variability impact mood and quality of life?. Diabetes Technol Ther.

[97] Critchley H.D., Garfinkel S.N. (2017). Interoception and emotion. Curr Opin Psychol.

[98] Nayok S.B., Sreeraj V.S., Shivakumar V., Venkatasubramanian G. (2023). A primer on interoception and its importance in psychiatry. Clin Psychopharmacol Neurosci.

[99] Quadt L., Critchley H.D., Garfinkel S.N. (2018). The neurobiology of interoception in health and disease. Ann N Y Acad Sci.

[100] Mehrhof S.Z., Fleming H., Nord C.L. (2025). An interoceptive model of energy allostasis linking metabolic and mental health. Sci Adv.

[101] Young H.A., Gaylor C.M., De Kerckhove D., Watkins H., Benton D. (2019). Interoceptive accuracy moderates the response to a glucose load: a test of the predictive coding framework. Proc Biol Sci.

[102] Zamariola G., Luminet O., Mierop A., Corneille O. (2019). Does it help to feel your body? Evidence is inconclusive that interoceptive accuracy and sensibility help cope with negative experiences. Cogn Emot.

[103] Barrett L.F., Quigley K.S., Bliss-Moreau E., Aronson K.R. (2004). Interoceptive sensitivity and self-reports of emotional experience. J Pers Soc Psychol.

[104] Banellis L., Nikolova N., Fischer Ehmsen J. (2025). Interoceptive ability is unrelated to mental health symptoms: evidence from a large scale multi-domain psychophysical investigation. Psychiatry Clin Psychology.

[105] Robinson E., Foote G., Smith J., Higgs S., Jones A. (2021). Interoception and obesity: a systematic review and meta-analysis of the relationship between interoception and BMI. Int J Obes.

[106] Khalsa S.S., Berner L.A., Anderson L.M. (2022). Gastrointestinal interoception in eating disorders: charting a new path. Curr Psychiatry Rep.

[107] Petzschner F.H., Garfinkel S.N., Paulus M.P., Koch C., Khalsa S.S. (2021). Computational models of interoception and body regulation. Trends Neurosci.

[108] Valente S., Di Girolamo G., Forlani M. (2017). Sex-specific issues in eating disorders: a clinical and psychopathological investigation. Eat Weight Disord.

[109] Füstös J., Gramann K., Herbert B.M., Pollatos O. (2013). On the embodiment of emotion regulation: interoceptive awareness facilitates reappraisal. Soc Cogn Affect Neurosci.

[110] Kusov P.A., Kotelevtsev Y.V., Drachev V.P. (2023). Cortisol monitoring devices toward implementation for clinically relevant biosensing in vivo. Molecules.

[111] Soares C.N., Poitras J.R., Prouty J. (2002). Hormone treatment for mood disorders in women. Expert Rev Neurother.

[112] Steiner M., Dunn E., Born L. (2003). Hormones and mood: from menarche to menopause and beyond. J Affect Disord.

[113] Kroemer N.B. (2023). Metabolic tuning during the menstrual cycle. Nat Metab.

[114] Hummel J., Benkendorff C., Fritsche L. (2023). Brain insulin action on peripheral insulin sensitivity in women depends on menstrual cycle phase. Nat Metab.

[115] Hengist A., Ong J.A., McNeel K., Guo J., Hall K.D. (2025). Imprecision nutrition? Intraindividual variability of glucose responses to duplicate presented meals in adults without diabetes. Am J Clin Nutr.

[116] Ali S., Stone M.A., Peters J.L., Davies M.J., Khunti K. (2006). The prevalence of co-morbid depression in adults with type 2 diabetes: a systematic review and meta-analysis. Diabet Med.

[117] Anderson R.J., Freedland K.E., Clouse R.E., Lustman P.J. (2001). The prevalence of comorbid depression in adults with diabetes. Diabetes Care.

[118] Jones B.D.M., Farooqui S., Kloiber S., Husain M.O., Mulsant B.H., Husain M.I. (2021). Targeting metabolic dysfunction for the treatment of mood disorders: review of the evidence. Life.

