# Supplementary material for: Glucose levels are associated with mood, but the association is mediated by ratings of metabolic state
Source: eBioMedicine. 2025 Dec 8;124:106035. doi: 10.1016/j.ebiom.2025.106035 (PMC12905624; doi:10.1016/j.ebiom.2025.106035)
Supplement: Supplementary Tables and Figures [file mmc1.docx]

# Supplementary Information

# Glucose levels are associated with mood, but the association is mediated by ratings of metabolic state

Kristin Kaduk^1,#^, Marie Kaeber^1,#^, Anne Kühnel^2^, María Berjano Torrado^1^, Melina Grahlow^1^, Birgit Derntl^1,3^, & Nils B. Kroemer^1-4*^

^#^ equal contribution

^1^ Department of Psychiatry and Psychotherapy, Tübingen Center for Mental Health, University of Tübingen, Tübingen, Germany

^2^ Section of Medical Psychology, Department of Psychiatry and Psychotherapy, Faculty of Medicine, University of Bonn, Bonn, Germany

^3^ German Center for Mental Health (DZPG), partner site Tübingen, Germany

^4^ German Center for Diabetes Research (DZD), Neuherberg, Germany

# Supplementary Material A: visualization of the Generalized Additive model (GAM) (related to Figure 4)


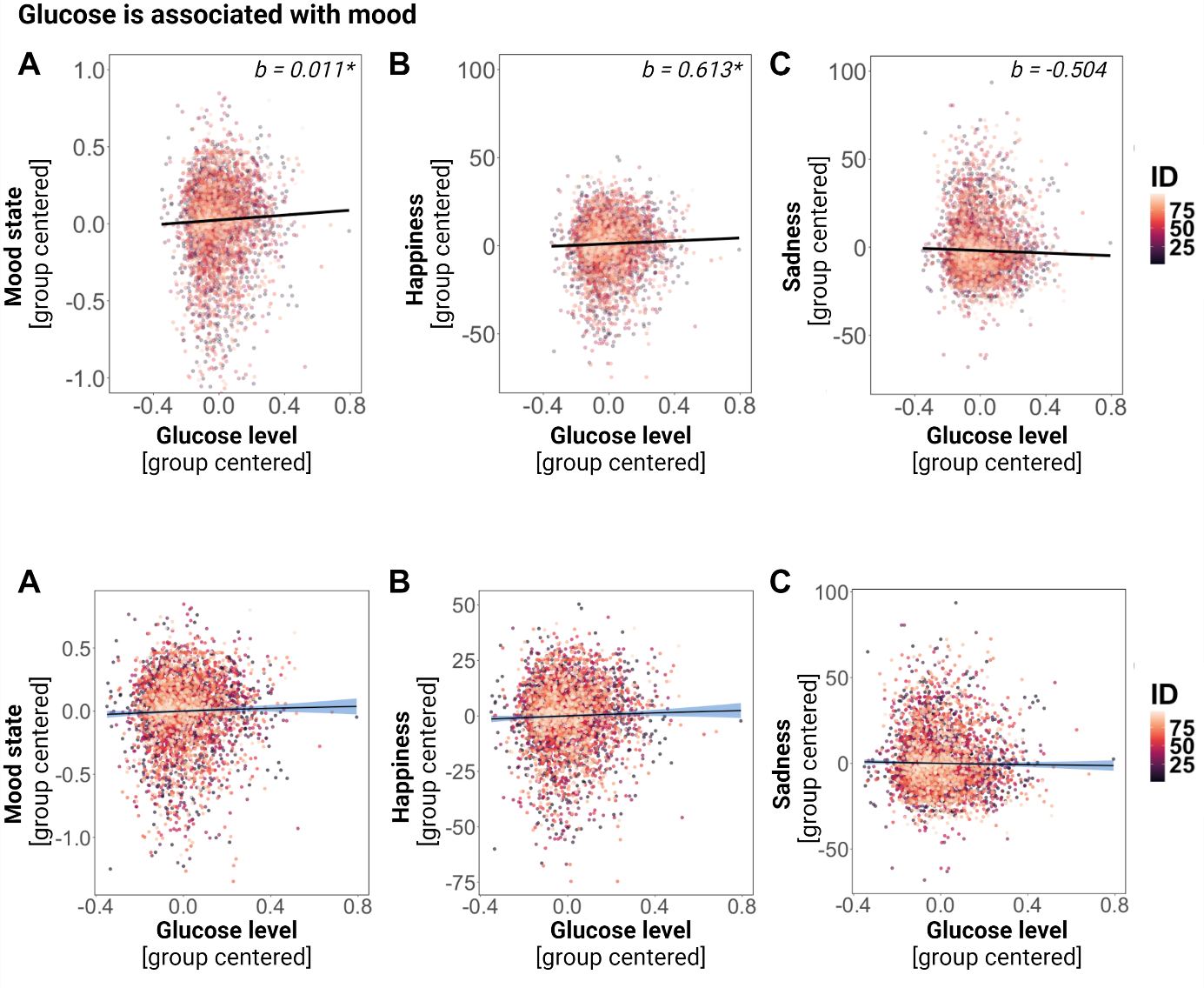


**Figure S1. Glucose levels are associated with mood and are independent of metabolic state.** The graphs display the relationship between glucose levels and **A:** mood state, **B:** happiness, and **C:** sadness, indicating that when glucose levels are high, participants reported higher mood. Blue lines indicate the relationship (fixed effect) across participants estimated by a Generalized Additive Model (GAM). We used a Generalized Additive Model (GAM) visualization to examine the non-linearity of the data, which confirmed that a linear model could adequately describe the relationship between glucose and mood. Each dot depicts an observation with a color-coding of participants. While mood state, happiness, and sadness are group centered on ID for visualization purposes, glucose level is log-transformed and group centered.

# Supplementary Material B: Distribution of the outcome variable mood state

**
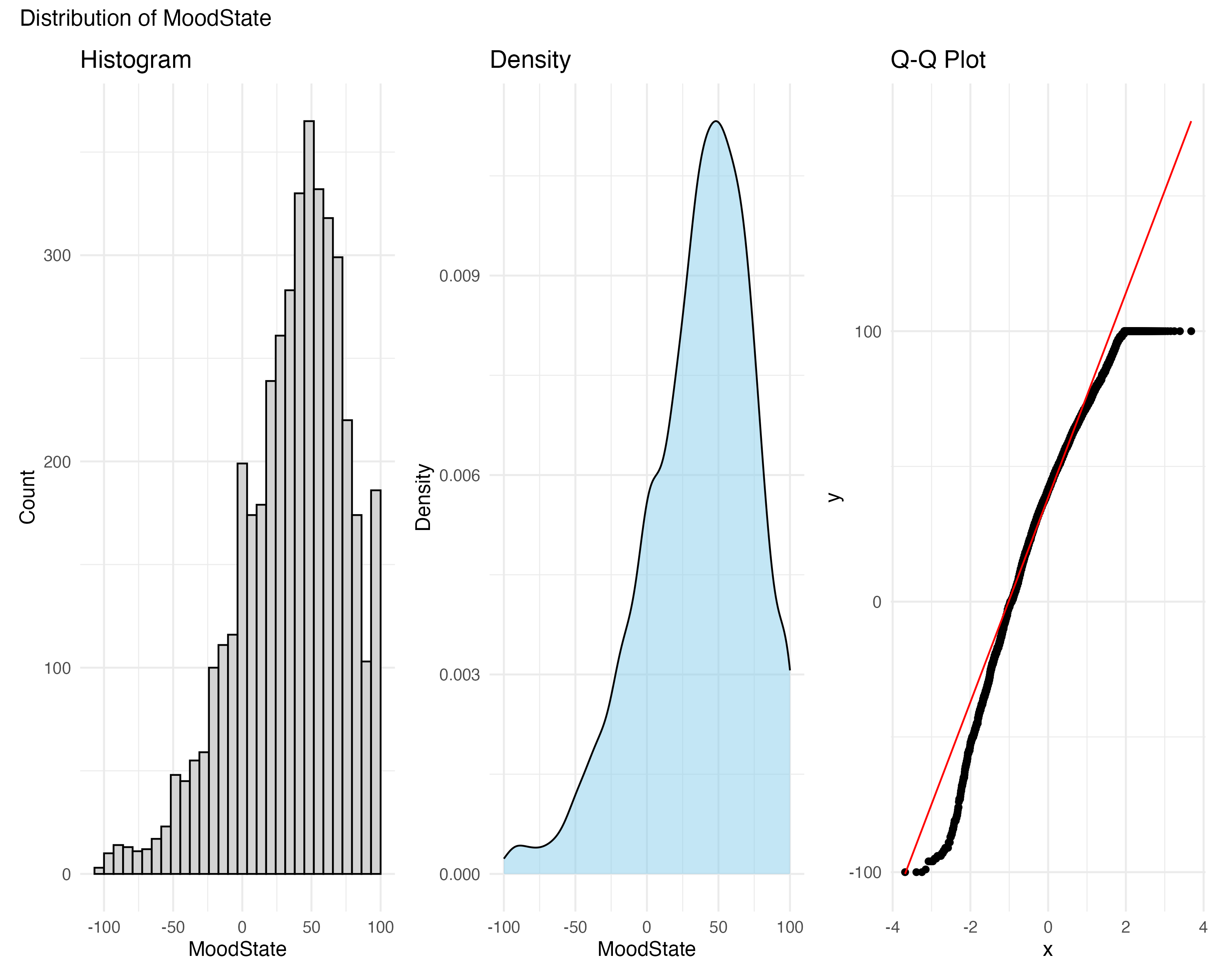
**

**Figure S2: Distribution of the outcome variable mood state.** The histogram (left) shows the frequency of MoodState values on the −100 to +100 scale, indicating a slightly left-skewed distribution with a peak around positive values. The kernel density estimate (middle) provides a smoothed representation of the same distribution. The corresponding quantile–quantile (Q–Q) plot (right) indicates moderate deviations from normality at the lower and upper tails.

# Supplementary Material C: Summary of the key assumptions of the LMEs and LMs

**Table S1. Summary of the diagnostics for the following models**

|  | **Mood state (1)** | **Metabolic state (2)** | **Mood state (3)** | **sdMood (5)** | **meanMood (5)** | **Interoceptive accuracy (4)** |
| --- | --- | --- | --- | --- | --- | --- |
| **Heteroscedasticity** | var. non-const. | var. non-const. | var. non-const. | Ok | Ok | var. non-const. |
| **Multicollinearity** | Low | Low | Low | Low | Low | Low |
| Distribution of residuals | non-normal (*p* < .001) | non-normal (*p* < .001) | non-normal (*p* < .001) | ok | non-normal (*p* = 0.021) | Ok |
| Overdispersion | No | No | No | - | - | - |
| **Outlier** or leverage points | Ok | Ok | Ok | Ok | Ok | Ok |

**Note.** Columns correspond to the following models: (1) mood state predicted from metabolic state and glucose levels, (2) metabolic state predicted from glucose levels, (3) mood state predicted from glucose levels, (4) interoceptive accuracy predicted from HOMA-IR, BMI, sex, and age, and (5) mean and standard deviation of mood state predicted from interoceptive accuracy. Rows report checks for **Heteroscedasticity (via Breusch-Pagan test), Multicollinearity (via Variance Inflation Factors), Distribution of residuals (via Shapiro-Wilk normality test), Overdispersion (via dispersion ratio test),** and **Outlier/leverage points using the appropriate functions from the performance package in R.**
var. non-const. = evidence of non-constant error variance; Ok = no issue detected; non-normal = residual normality test result; Low (multicollinearity) = low predictor collinearity; No (overdispersion) = dispersion near 1; “–” = not applicable.

**
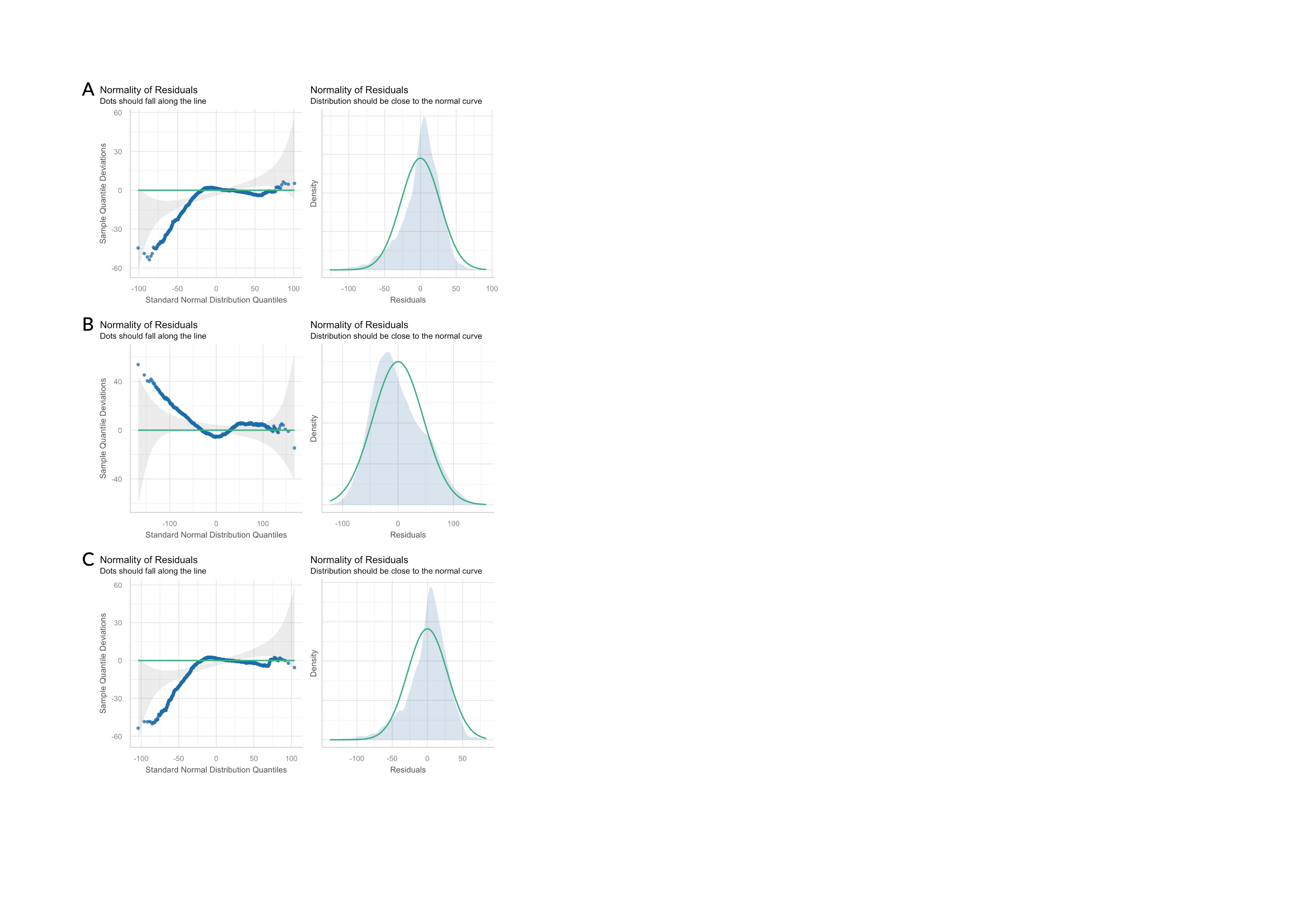
**

**Figure S3. Normality checks of studentized residuals from the LMEs.** **A:** Mood state predicted by glucose levels and metabolic state, **B:** Metabolic state predicted by glucose levels, **C:** Mood state predicted by glucose levels. The quantile–quantile (Q–Q) plots (left panels) compare the distribution of studentized residuals against the theoretical quantiles of a standard normal distribution, with shaded simulation bands indicating the expected range under normality. Departures from the horizontal line outside the bands reflect deviations from normality, particularly in the tails. The density plots (right panels) show the empirical distribution of studentized residuals (blue area) overlaid with the standard normal curve (green line), indicating approximate symmetry but heavier tails in some models.

# Supplementary Material D: Wild bootstrapping

**Table S2.** Parameter estimates from linear mixed-effects models with parametric and wild bootstrap inference.

| \| Term \| Method \| Estimate \| SE \| \| **95% CI Lower** \| \| --- \| \| \| **95% CI Upper** \| \| --- \| \| \| --- \| --- \| --- \| --- \| --- \| --- \| --- \| --- \| \| MoodState ~ z.MetState * z.logGlu * (z.res_log_HOMA + z.BMI + cSex + z.Age) + (1 + z.MetState * z.logGlu\|ID) \| \| \| \| \| \| \| Intercept \| Parametric \| 35.894 \| 2.691 \| 30.702 \| 41.068 \| \| Intercept \| Wild Bootstrap \| 35.894 \| 2.520 \| 31.056 \| 40.873 \| \| z.logGlu \| Parametric \| 0.356 \| 0.636 \| -0.890 \| 1.560 \| \| z.logGlu \| Wild Bootstrap \| 0.356 \| 0.648 \| -0.836 \| 1.683 \| \| z.MetState \| Parametric \| -2.953 \| 0.686 \| -4.298 \| -1.633 \| \| z.MetState \| Wild Bootstrap \| -2.953 \| 0.648 \| -4.197 \| -1.681 \| \| z.MetState:z.logGlu \| Parametric \| 0.729 \| 0.617 \| -0.481 \| 1.913 \| \| z.MetState:z.logGlu \| Wild Bootstrap \| 0.729 \| 0.622 \| -0.469 \| 1.998 \| \| MetState ~ z.logGlu * (z.res_log_HOMA + z.BMI + cSex + z.Age) + (1 + z.logGlu\|ID) \| \| \| \| \| \| \| Intercept \| Parametric \| -27.588 \| 1.929 \| -31.284 \| -23.848 \| \| Intercept \| Wild Bootstrap \| -27.588 \| 1.821 \| -31.153 \| -24.198 \| \| z.logGlu \| Parametric \| -17.879 \| 1.022 \| -19.825 \| -15.886 \| \| z.logGlu \| Wild Bootstrap \| -17.879 \| 0.972 \| -19.826 \| -16.028 \| \| MoodState ~ z.logGlu * ( z.res_log_HOMA + cSex + z.BMI + z.Age) + (1 + z.logGlu\|ID) \| \| \| \| \| \| \| Intercept \| Parametric \| 35.946 \| 2.748 \| 30.637 \| 41.226 \| \| Intercept \| Wild Bootstrap \| 35.946 \| 2.533 \| 31.073 \| 40.874 \| \| z.logGlu \| Parametric \| 1.135 \| 0.537 \| 0.092 \| 2.165 \| \| z.logGlu \| Wild Bootstrap \| 1.135 \| 0.534 \| 0.067 \| 2.159 \| |
| --- | --- | --- | --- | --- | --- | --- | --- | --- | --- | --- | --- | --- | --- | --- | --- | --- | --- | --- | --- | --- | --- | --- | --- | --- | --- | --- | --- | --- | --- | --- | --- | --- | --- | --- | --- | --- | --- | --- | --- | --- | --- | --- | --- | --- | --- | --- | --- | --- | --- | --- | --- | --- | --- | --- | --- | --- | --- | --- | --- | --- | --- | --- | --- | --- | --- | --- | --- | --- | --- | --- | --- | --- | --- | --- | --- | --- | --- | --- | --- | --- | --- | --- | --- | --- | --- | --- | --- | --- | --- | --- | --- | --- | --- | --- | --- | --- | --- | --- | --- | --- | --- | --- | --- | --- | --- | --- | --- | --- | --- | --- | --- | --- | --- | --- | --- | --- | --- | --- | --- | --- | --- | --- |

**Note.** Columns report fixed‐effect parameter estimates (Estimate), standard errors (SE), and 95% confidence intervals for each model term. Estimates are presented for both conventional parametric inference and wild bootstrap inference, which yielded highly similar results.

# Supplementary Material E: Sensitivity analyses (BMI < 30, HOMA-IR < 2.5)

**Table S3. Sensitivity analyses comparing mediation effects (ACME, ADE, Proportion Mediated) across the full, BMI < 30, and HOMA-IR < 2.5 samples.**

| **Metric** | **Sample** | **ACME (average)** | **ADE (average)** | **Proportion Mediated (average)*** |
| --- | --- | --- | --- | --- |
| **Estimate** | Baseline | 0.775 | 0.333 | 0.680 |
|  | BMI<30 | 0.868 | 0.193 | 0.782 |
|  | HOMA<2.5 | 0.796 | 0.053 | 0.833 |
| **Lower 95% CI** | Baseline | 0.331 | -0.747 | 0.172 |
|  | BMI<30 | 0.342 | -0.974 | -1.824 |
|  | HOMA<2.5 | 0.289 | -1.113 | -4.966 |
| **Upper 95% CI** | Baseline | 1.224 | 1.428 | 3.941 |
|  | BMI<30 | 1.401 | 1.385 | 5.775 |
|  | HOMA<2.5 | 1.305 | 1.255 | 8.046 |
| ***p*-value** | Baseline | 0.001 | 0.544 | 0.039 |
|  | BMI<30 | 0.001 | 0.746 | 0.063 |
|  | HOMA<2.5 | 0.010 | 0.926 | 0.133 |

*****A proportion mediated is a ratio of indirect/total effect, and it can fall outside the [0,1] due to estimation uncertainty or opposing directions of effect.

**Table S4. Sensitivity analyses comparing effects of the LME for Moodstate – Glucose levels * MetState across the full, BMI < 30, and HOMA-IR < 2.5 samples.**

| **Model** | **Term** | **Estimate** | ***p*-value** | **95% CI Lower** | **95% CI Upper** | **star** |
| --- | --- | --- | --- | --- | --- | --- |
| **MoodState ~ z.MetState * z.logGlu * (z.res_log_HOMA + z.BMI + cSex + z.Age) + (1 + z.MetState * z.logGlu\|ID)** | | | | | | |
| Baseline | Intercept | 35.894 | <0.001 | 30.702 | 41.068 | *** |
|  | z.logGlu | 0.356 | 0.577 | -0.890 | 1.560 |  |
|  | z.MetState | -2.953 | <0.001 | -4.298 | -1.633 | *** |
|  | z.MetState:z.logGlu | 0.729 | 0.241 | -0.481 | 1.913 |  |
|  | z.MetState:z.logGlu:cSex | 3.062 | 0.015 | 0.679 | 5.480 | * |
| HOMA<2.5 | Intercept | 38.077 | <0.001 | 31.942 | 44.191 | *** |
|  | z.logGlu | 0.044 | 0.953 | -1.393 | 1.444 |  |
|  | z.MetState | -2.822 | <0.001 | -4.281 | -1.402 | *** |
|  | z.MetState:z.logGlu | 0.488 | 0.486 | -0.858 | 1.839 |  |
|  | z.MetState:z.logGlu:cSex | 3.800 | 0.004 | 1.296 | 6.357 | ** |
| BMI<30 | Intercept | 38.527 | <0.001 | 32.716 | 44.325 | *** |
|  | z.logGlu | 0.056 | 0.939 | -1.348 | 1.414 |  |
|  | z.MetState | -3.431 | <0.001 | -4.952 | -1.934 | *** |
|  | z.MetState:z.logGlu | 0.824 | 0.253 | -0.567 | 2.202 |  |
|  | z.MetState:z.logGlu:cSex | 3.570 | 0.009 | 1.002 | 6.221 | ** |
| **MoodState ~ z.logGlu * (z.res_log_HOMA + z.BMI + cSex + z.Age) + (1 + z.logGlu\|ID)** | | | | | | |
| Baseline | Intercept | 35.946 | <0.001 | 30.637 | 41.226 | *** |
|  | z.logGlu | 1.135 | 0.038 | 0.092 | 2.165 | * |
| HOMA<2.5 | Intercept | 38.179 | <0.001 | 31.924 | 44.397 | *** |
|  | **z.logGlu** | **1.074** | **0.093** | **-0.145** | **2.283** |  |
| BMI<30 | Intercept | 38.815 | <0.001 | 32.880 | 44.714 | *** |
|  | **z.logGlu** | **1.033** | **0.099** | **-0.161** | **2.221** |  |
| **Metstate ~ z.logGlu * (z.res_log_HOMA + z.BMI + cSex + z.Age) + (1 + z.logGlu\|ID)** | | | | | | |
| Baseline | Intercept | -27.588 | <0.001 | -31.284 | -23.848 | *** |
|  | z.logGlu | -17.879 | <0.001 | -19.825 | -15.886 | *** |
| HOMA<2.5 | Intercept | -28.340 | <0.001 | -32.779 | -23.868 | *** |
|  | z.logGlu | -18.435 | <0.001 | -20.633 | -16.180 | *** |
| BMI<30 | Intercept | -27.264 | <0.001 | -31.660 | -22.822 | *** |
|  | z.logGlu | -18.211 | <0.001 | -20.473 | -15.892 | *** |


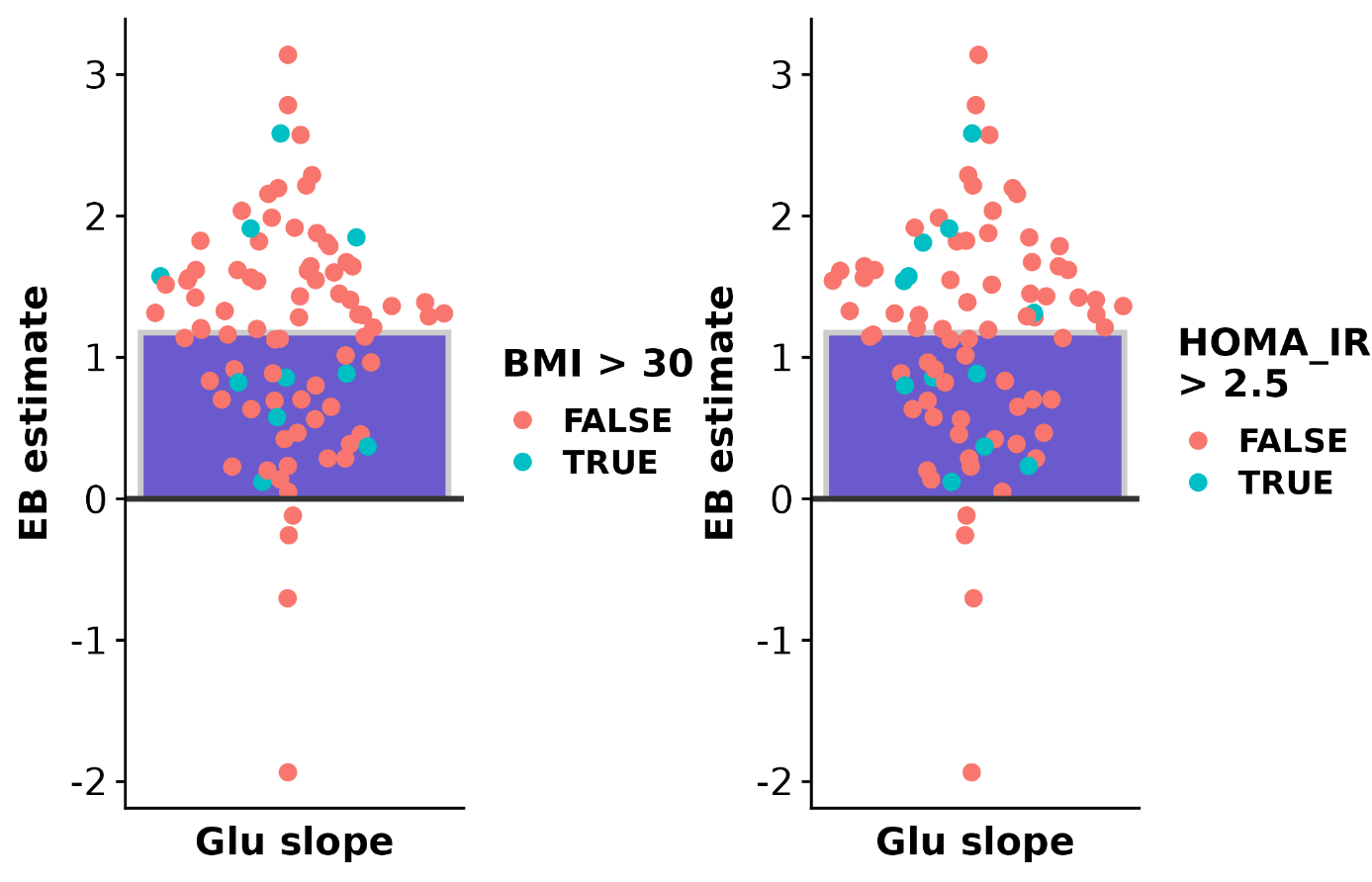


**Figure S4.** **Individual glucose–mood slopes are comparable across BMI and HOMA-IR subgroups.** The association of glucose levels with mood is similar in participants with higher BMI and indicators of insulin resistance (BMI > 30 or HOMA-IR > 2.5). **(Left panel)** The individual slopes of the current glucose levels predicting the current mood state are similar in participants with a BMI > 30 vs. < 30 (t(11)=0.08, *p*=.93). The slopes from participants with a BMI > 30 are distributed around the average slope across participants. **(Right panel)** Individual slopes predicting mood state by the current glucose levels do not differ in participants with indications of insulin resistance (t(15)=0.04, *p*=.96). The empirical Bayes estimates (EB) are the individual slope coefficients extracted from the model predicting MoodState by z-standardized glucose levels without accounting for interindividual differences in the model: MoodState ~ z.logGlu + (1 + z.logGlu |ID)).

# Supplementary Material F: Results with menstrual cycle phase

**
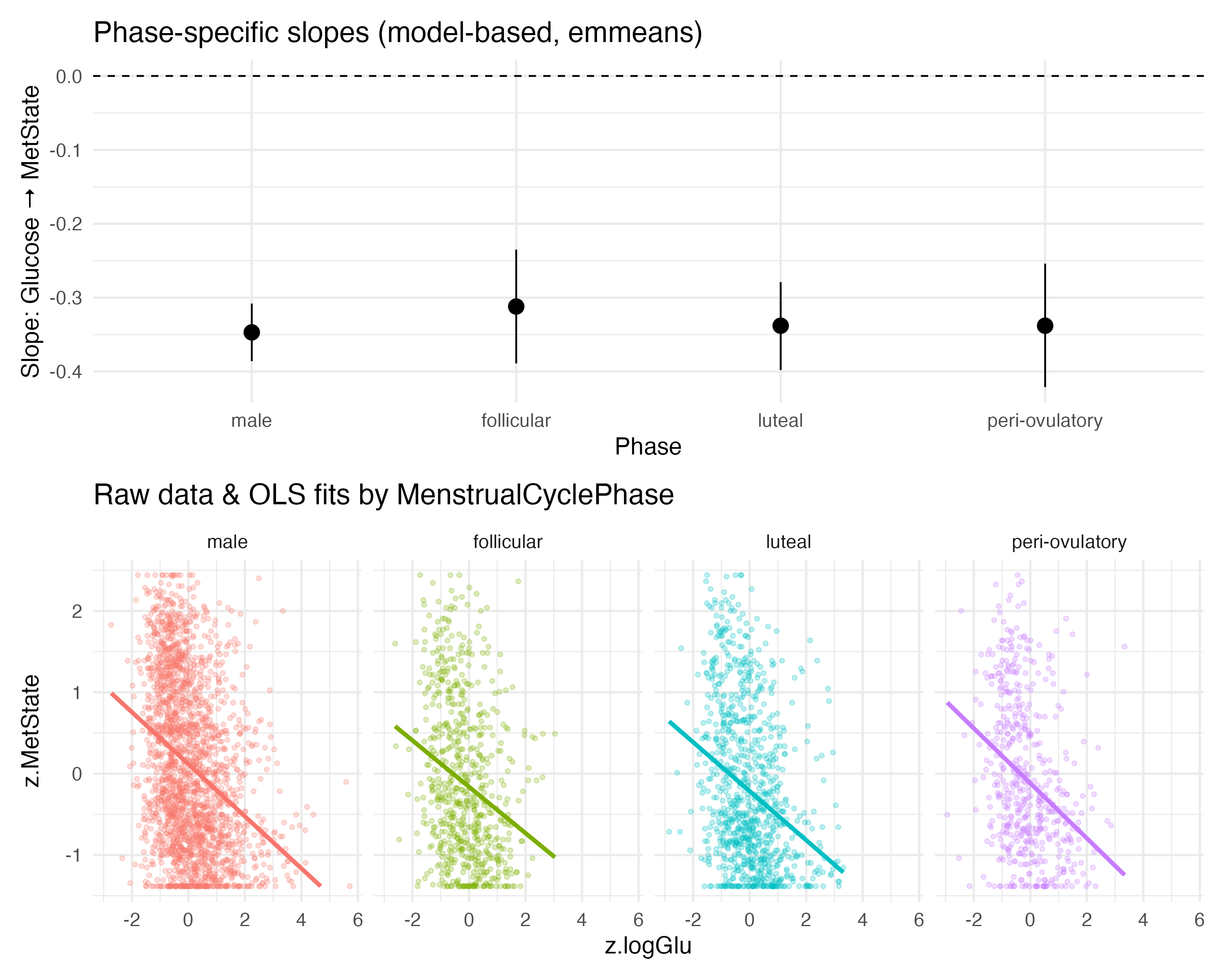
**

**Figure S5. No phase-specific difference in the negative association between glucose and metabolic state. A:** Model-based slopes from the **LME**, z.MetState *~* z.logGlu *** **MenstrualCyclePhase** + (1|ID). Points are **phase**-specific estimates of the slope of metabolic state on glucose; error bars are 95% CIs; the dashed line marks zero. Asterisks indicate significant pairwise differences between **phase**-specific slopes (emmeans/emtrends, multiplicity-adjusted). **B:** Raw data by **phase** with robust linear fit shown for visualization only; axes display z-scored variables, and panels correspond to male, follicular, luteal, and peri-ovulatory groups. Random intercepts for participants were included; no additional covariates were entered.

**Table S5 Model output for the MenstrualCyclePhase-coded Model**

| **Names** | **Estimate** | **Std.Error** | **df** | **t.value** | **Pvalue** | **star** |
| --- | --- | --- | --- | --- | --- | --- |
| (Intercept) | 37.320 | 3.740 | 89 | 9.98 | <0.001 | *** |
| **z.MetState** | -2.095 | 0.943 | 96 | -2.22 | 0.029 | * |
| z.logGlu | 0.666 | 0.843 | 71 | 0.79 | 0.432 |  |
| z.res_log_HOMA | -0.024 | 2.564 | 90 | -0.01 | 0.993 |  |
| z.BMI | 1.177 | 2.733 | 89 | 0.43 | 0.668 |  |
| z.Age | -0.170 | 2.804 | 89 | -0.06 | 0.952 |  |
| phaseFollicular | -2.282 | 5.366 | 96 | -0.43 | 0.672 |  |
| phaseLuteal | -2.993 | 5.333 | 93 | -0.56 | 0.576 |  |
| phasePeri-ovulatory | -3.433 | 5.407 | 99 | -0.63 | 0.527 |  |
| **z.MetState:z.logGlu** | 2.263 | 0.813 | 99 | 2.78 | 0.006 | ** |
| z.MetState:z.res_log_HOMA | -0.143 | 0.684 | 120 | -0.21 | 0.835 |  |
| z.MetState:z.BMI | -0.033 | 0.704 | 102 | -0.05 | 0.963 |  |
| z.MetState:z.Age | -0.562 | 0.729 | 103 | -0.77 | 0.442 |  |
| z.MetState:phaseFollicular | -0.495 | 1.696 | 236 | -0.29 | 0.771 |  |
| z.MetState:phaseLuteal | -1.122 | 1.560 | 174 | -0.72 | 0.473 |  |
| **z.MetState:phasePeri-ovulatory** | -4.697 | 1.856 | 313 | -2.53 | 0.012 | * |
| z.logGlu:z.res_log_HOMA | 0.630 | 0.605 | 89 | 1.04 | 0.300 |  |
| z.logGlu:z.BMI | 0.262 | 0.692 | 109 | 0.38 | 0.706 |  |
| z.logGlu:z.Age | -0.368 | 0.649 | 82 | -0.57 | 0.572 |  |
| z.logGlu:phaseFollicular | -0.640 | 1.643 | 233 | -0.39 | 0.697 |  |
| z.logGlu:phaseLuteal | -0.199 | 1.470 | 152 | -0.14 | 0.892 |  |
| z.logGlu:phasePeri-ovulatory | -1.769 | 1.785 | 287 | -0.99 | 0.323 |  |
| z.MetState:z.logGlu: z.res_log_HOMA | 0.788 | 0.610 | 139 | 1.29 | 0.198 |  |
| z.MetState:z.logGlu:z.BMI | 0.690 | 0.681 | 153 | 1.01 | 0.312 |  |
| z.MetState:z.logGlu:z.Age | -0.267 | 0.636 | 123 | -0.42 | 0.675 |  |
| z.MetState:z.logGlu:phasefollicular | -2.868 | 1.677 | 390 | -1.71 | 0.088 |  |
| **z.MetState:z.logGlu: phaseluteal** | -2.797 | 1.412 | 203 | -1.98 | 0.049 | * |
| **z.MetState:z.logGlu: phaseperiovulatory** | -4.007 | 1.802 | 427 | -2.22 | 0.027 | * |

**Note.** Results from the linear mixed-effects model predicting mood state from metabolic state, glucose levels, insulin resistance (HOMA-IR), BMI, sex, and age, with menstrual cycle phase coded as categorical predictors (reference category: male). Fixed-effect estimates (Estimate), standard errors (Std. Error), denominator degrees of freedom (df), t-values, and p-values are reported. Interaction terms with menstrual cycle phase indicate whether the associations between metabolic state, glucose, and mood differed across follicular, luteal, and peri-ovulatory phases relative to the male reference group.

**Table S6 Phase-specific coefficients**

| **Phase** | **z.logGlu.trend** | **SE** | **df** | **lower.CL** | **upper.CL** |
| --- | --- | --- | --- | --- | --- |
| male | -0.347 | 0.020 | 4285 | -0.386 | -0.308 |
| follicular | -0.312 | 0.039 | 4262 | -0.389 | -0.235 |
| luteal | -0.338 | 0.030 | 4271 | -0.398 | -0.279 |
| peri-ovulatory | -0.338 | 0.042 | 4264 | -0.421 | -0.254 |

**Note.** Phase-specific slopes of the effect of glucose on metabolic state from the linear mixed-effects model z.MetState ~ z.logGlu * Z_phase + (1|ID). Reported are the estimated slope (z.logGlu.trend), standard error (SE), degrees of freedom (df), and 95% confidence intervals (lower.CL, upper.CL). Negative values indicate that higher glucose levels are associated with a lower (hungrier) metabolic state. Phases: male (reference), follicular, luteal, peri-ovulatory.

# Supplementary Material G: Analyses of the covariate, sleep duration

**Table S7. Sensitivity analyses comparing the effects of the LME for MoodState – Glucose levels * MetState across when including sleep duration.**

| Model | Term | Estimate | *p*-value | 95% CI Lower | 95% CI Upper | star |
| --- | --- | --- | --- | --- | --- | --- |
| MoodState ~ z.MetState * z.logGlu * (z.res_log_HOMA + z.BMI + cSex + z.Age) + (1 + z.MetState * z.logGlu\|ID) | | | | | | |
| Baseline | Intercept | 35.894 | <0.001 | 30.702 | 41.068 | *** |
|  | z.logGlu | 0.356 | 0.577 | -0.890 | 1.560 |  |
|  | z.MetState | -2.953 | <0.001 | -4.298 | -1.633 | *** |
|  | z.MetState:z.logGlu | 0.729 | 0.241 | -0.481 | 1.913 |  |
|  | z.MetState:z.logGlu:cSex | 3.062 | 0.015 | 0.679 | 5.480 | * |
| Including sleep duration | Intercept | 35.754 | <0.001 | 30.425 | 41.067 | *** |
|  | z.logGlu | 0.466 | 0.475 | 0.801 | 1.677 |  |
|  | z.MetState | -2.955 | <0.001 | 4.354 | -1.586 | *** |
|  | z.MetState:z.logGlu | 0.692 | 0.274 | -0.528 | 1.895 |  |
|  | z.MetState:z.logGlu:cSex | 3.299 | 0.010 | 0.914 | 5.746 | ** |
| MoodState ~ z.logGlu * (z.res_log_HOMA + z.BMI + cSex + z.Age) + (1 + z.logGlu\|ID) | | | | | | |
| Baseline | Intercept | 35.946 | <0.001 | 30.637 | 41.226 | *** |
|  | z.logGlu | 1.135 | 0.038 | 0.092 | 2.165 | * |
| Including sleep duration | Intercept | 38.179 | <0.001 | 30.379 | 41.246 | *** |
|  | z.logGlu | 1.232 | 0.027 | 0.173 | 2.270 |  |

# Supplementary Material H: Additional analyses to test covariates

**Table S8. Model comparison for the inclusion of covariates.**

| Model | par | AIC | BIC | logLik | deviance | χ² | Df | *p*-value |
| --- | --- | --- | --- | --- | --- | --- | --- | --- |
| Baseline | 31 | 40201 | 40398 | -20069.6 | 40139 |  |  |  |
| Student status | 35 | 40205 | 40426 | -20067.3 | 40135 | 4.66 | 4 | 0.324 |
| IPAQ | 35 | 40206 | 40428 | -20068.0 | 40136 | 3.11 | 4 | 0.540 |
| FTND | 35 | 40202 | 40424 | -20066.1 | 40132 | 6.88 | 4 | 0.142 |
| AUDIT | 35 | 40208 | 40430 | -20068.8 | 40138 | 1.49 | 4 | 0.828 |
| Sleep duration | 35 | 40207 | 40429 | 20068.0 | 40137 | 2.09 | 4 | 0.072 |
| Menstrual cycle phase | 39 | 41341 | 41589 | -20631.0 | 41263 | 5.85 | 8 | 0.663 |
| Assessment time | 40 | 40198 | 40451 | -20058.8 | 40118 | 15.57 | 9 | 0.010 |

**Note.** Likelihood ratio tests were conducted to evaluate whether adding covariates (student status, physical activity assessed via IPAQ, smoking behavior assessed via FTND, and alcohol use assessed via AUDIT, sleep duration, and menstrual cycle phase via self-report, assessment time) improved model fit relative to the baseline model. For each comparison, the table reports the number of parameters (npar), model fit indices (AIC, BIC, log-likelihood, deviance), Chisq (*χ*²) test statistics, associated degrees of freedom, and *p*-values.

# Supplementary Material I: Analyses of the covariate, assessment time

To evaluate whether the covariate, assessment time, influenced the associations of glucose and metabolic state with mood, we added time as a linear predictor. A model comparison showed a better likelihood fit when including assessment time (χ²(9) = 21.64, *p* = .010), although the BIC increased (40398 vs. 40451; ΔBIC = 53). Inspection of residuals confirmed that modeling assessment time as a linear predictor adequately captured the temporal trend (Figure S6).

Mood declined over the day (Time, *b* = -1.434, *t*(78) = -2.48, 95% CI [-2.604, -0.298], *p* = .015) and the glucose effect varied by time (Glucose × Time, *b* = -1.194, *t*(1629) = -2.26, 95% CI [−2.245, −0.148], *p* = .024), showing a stronger positive association with mood in the morning that diminished toward the afternoon. Moreover, we observed a significant three-way interaction, indicating that the interaction of glucose levels and metabolic state was moderated by assessment time (MetState × Glucose × Time, *b* = -1.079, *t*(2881) = -1.98, 95% CI [−2.150, −0.007], *p* = .048).

To sum up, including assessment time yielded small but significant interactions with glucose and metabolic state; however, it did not alter our main findings or conclusions. Thus, assessment time is not driving the reported associations, although it explains some of the observed variability in the estimates.

**
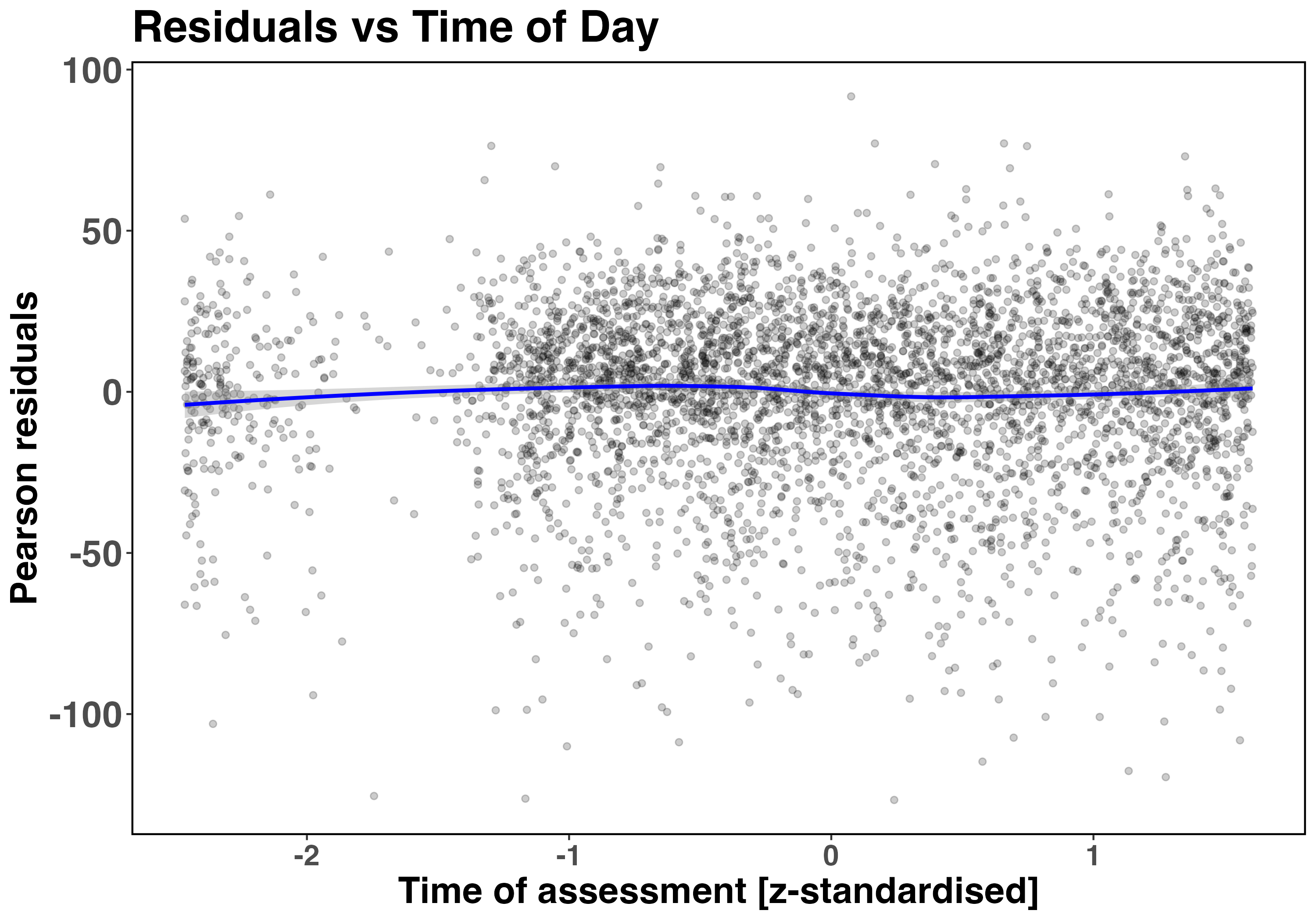
**

**Figure S6.** Residual plot of the LME against time of assessment. Each point represents the residual of a single observation, and the blue line shows a LOESS smoother. The residuals are evenly scattered around zero without systematic curvature, indicating that the linear specification of time adequately captured the temporal trend.
